# Macrophage PABPC4‐SPP1 Axis Orchestrates Immunosuppression in Colorectal Cancer

**DOI:** 10.1002/advs.77361

**Published:** 2026-08-21

**Authors:** Meng Wang, Fang Yang, Yuan Gao, Liuli Li, Yulan Huang, Hui Yao, Sijie Zhao, Kun Zhao, Tianying Zhang, Hui Heng, Jiaxin Xu, Yuancai Xiang, Weidong Xiao, Shaofan Hu, Hongming Miao

**Affiliations:** ^1^ Department of Pathophysiology College of High Altitude Military Medicine Third Military Medical University (Army Medical University) Chongqing China; ^2^ Jinfeng Laboratory Chongqing China; ^3^ Key Laboratory of Extreme Environmental Medicine Ministry of Education of China Third Military Medical University (Army Medical University) Chongqing China; ^4^ Department of Physiology School of Basic Medical Sciences Southwest Medical University Luzhou Sichuan China; ^5^ Department of Biochemistry and Molecular Biology School of Basic Medical Sciences Southwest Medical University Luzhou Sichuan China; ^6^ Department of General Surgery the Second Affiliated Hospital of Army Medical University Chongqing China

**Keywords:** CD8^+^ T cell, colorectal cancer, macrophage, PABPC4, SPP1

## Abstract

Poly(A)‐binding proteins (PABPs) are frequently dysregulated in cancers, yet their functional roles remain largely elusive. Here, we identify that PABPC4, a member of PABPs, is upregulated during colorectal cancer (CRC) progression and associated with poor prognosis. Single‐cell transcriptomics reveals that PABPC4 is predominantly expressed in the macrophages of the tumor microenvironment (TME). Conditional knockout of *Pabpc4* in macrophages (C4cKO) profoundly inhibited the progression of multiple cancers, including CRC, melanoma, and ovarian cancer. Mechanistically, C4cKO obviously enhances the local and systemic antitumor immunity, characterized by increased M1‐like macrophages and cytotoxic CD8^+^ T cells, and reduced M2‐like macrophages and myeloid‐derived suppressor cells. Clearance of macrophages or CD8^+^ T cells could abolish C4cKO‐stimulated antitumor immunity. We further demonstrate that PABPC4 stabilizes *SPP1* mRNA via direct 3′UTR binding, and SPP1 restoration substantially reversed the enhanced antitumor immunity and tumor suppression in C4cKO mice. Clinically, elevated macrophage PABPC4‐SPP1 axis activity in human CRC tissues correlates with immunosuppressive phenotypes, potential immunotherapy resistance, and poor survival of patients. Collectively, our results reveal a previously undiscovered PABPC4‐SPP1 axis in macrophages, which induces tumor‐promoting macrophages and CD8^+^ T cell dysfunction, thereby facilitating tumor progression. Targeting the PABPC4‐SPP1 axis represents a promising strategy for cancer therapy.

## Graphical abstract

In the CRC microenvironment, macrophage PABPC4 binds to the 3′UTR of *SPP1* mRNA to stabilize its expression, thereby sustaining M2‐like immunosuppressive macrophage polarization. Concurrently, this axis suppresses CD8^+^ T cell effector functions via CD44 signaling, collectively fostering an immunosuppressive niche that drives tumor progression.

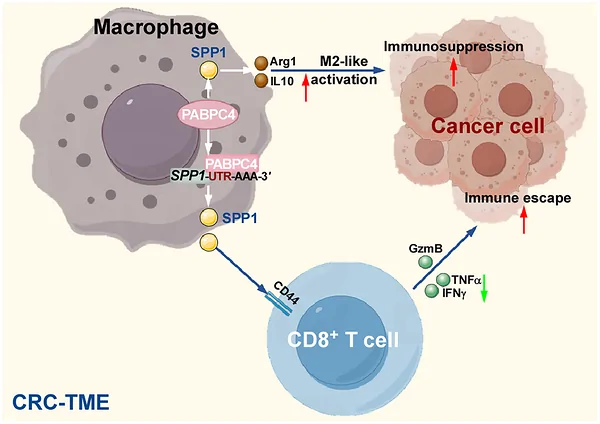

## 1 Introduction

As a highly prevalent and lethal cancer worldwide, colorectal cancer (CRC) is largely shaped by an immunosuppressive tumor microenvironment (TME) that promotes disease advancement and diminishes treatment effectiveness [1, 2]. Within this setting, macrophages emerge as a predominant and functionally plastic immune component, whose abundance and polarization state are strongly associated with clinical outcome [2, 3]. They are frequently co‐opted by the tumor, undergoing a functional repolarization from tumor‐suppressive (M1‐like) to pro‐tumorigenic (M2‐like) phenotypes, thereby becoming tumor‐associated macrophages (TAMs) [4]. These M2‐like TAMs drive disease advancement through multifaceted mechanisms, including direct support of tumor growth and metastasis, enforcement of an immunosuppressive niche, and participation in metabolic reprogramming [4, 5]. Consequently, therapeutic strategies aimed at modulating TAM function have gained considerable focus in oncology research [5]. However, translating these approaches into effective clinical interventions requires a more comprehensive mechanistic elucidation of the specific molecular pathways that govern TAM behavior within the TME.

RNA‐binding proteins (RBPs) serve as key arbiters of gene expression at the post‐transcriptional level, governing critical steps such as splicing, mRNA decay, intracellular transport, and translational control to fine‐tune functional genomic output [6]. Their dysregulation disrupts RNA homeostasis, leading to aberrant expression of oncogenic and immunosuppressive programs that fuel malignancy, therapy resistance, and immune evasion [7, 8]. Although RBPs are increasingly recognized as contributors to tumorigenesis and as potential therapeutic targets [9, 10], their cell type‐specific roles and precise mechanisms of action within the complex TME remain poorly defined.

Poly(A)‐binding proteins (PABPs) constitute an important RBP subfamily that critically regulates translation initiation, mRNA stability, and subcellular localization [11, 12]. Comprising nuclear (e.g., PABPN1) and cytoplasmic (e.g., PABPC1, PABPC4) members, PABPs recognize mRNA poly(A) tails or specific 3′UTR sequences via conserved RNA recognition motifs (RRMs) [11, 13]. Emerging evidence indicates that several PABP members, including PABPC1, PABPC3, PABPC4, and PABPN1, are aberrantly expressed across cancer types and can influence tumor progression by modulating target gene expression in cancer cells [13, 14, 15, 16]. Nevertheless, their functions and mechanisms within the CRC TME, particularly in stromal immune cells such as TAMs, have yet to be fully characterized.

Here, we pinpoint PABPC4 as a poly(A)‐binding protein linked to adverse prognosis in CRC. We demonstrate that elevated PABPC4 expression in macrophages fosters an immunosuppressive TME and accelerates tumor progression by stabilizing *SPP1* mRNA, thereby suppressing CD8^+^ T cell activity. The functional relevance of the macrophage PABPC4‐SPP1 axis was validated through genetic ablation and rescue experiments in vivo, and its activity correlates with advanced pathological features in CRC patients. These findings delineate a previously unrecognized post‐transcriptional pathway that orchestrates immunosuppressive polarization of macrophages, positioning this axis as a viable candidate for colorectal cancer immunotherapy.

## 2 Materials and Methods

### 2.1 Reagents

Dimethyl sulfoxide (DMSO; #D8371), lipopolysaccharides (LPS; #L8880), EDTA (#E1170), and hyaluronidase (#H8030) were obtained from Solarbio (Beijing, China). Collagenase IV (#2091GR001) was sourced from BioFroxx (Hesse, Germany). DNase I (#10104159001) was acquired from Merck Millipore (MA, USA). Recombinant mouse IL‐4 (#574304), anti‐mouse CD3ε (#100340; RRID: AB_11149115), recombinant mouse M‑CSF (carrier‑free) (#576406), Cell Activation Cocktail (with Brefeldin A) (#423303) and anti‐mouse CD28 (#102116; RRID: AB_11147170) were purchased from BioLegend (CA, USA). Recombinant Murine IL‐2 (#AF‐212‐12) was acquired from PeproTech (NJ, USA). D‑Luciferin potassium salt (#ST196) was supplied by Beyotime (Shanghai, China). Thioglycolate medium (#02‐015) was obtained from AOBOX (Beijing, China). Azoxymethane (AOM; #A5486) was acquired from Sigma‑Aldrich (MO, USA). Dextran sodium sulfate (DSS; #160110) was sourced from MP Biomedicals (CA, USA). Actinomycin D (ActD; #HY‑17559) was provided by MedChemExpress (MCE, Shanghai, China). Rat IgG2b isotype control‑InVivo (#A2116; RRID: AB_3662740), anti‑mouse CD4‑InVivo (#A2101; RRID: AB_3677296), anti‑mouse CD8α‑InVivo (#A2102; RRID: AB_3099521), rat IgG2a isotype control‐InVivo (#A2123; RRID: AB_3644245) and anti‐mouse PD‐1 (CD279)‐InVivo (#A2122; RRID: AB_3644244) were obtained from Selleck (TX, USA). PBS liposomes (#K2722) and clodronate liposomes (#K2721) were purchased from APExBIO (TX, USA). Recombinant Mouse SPP1 protein (#RP02806) was acquired from Abclonal (Wuhan, China). Rat IgG2b kappa isotype control (#14‐4031‐81; RRID: AB_470098), CD44 monoclonal antibody (#14‐0441‐81; RRID: AB_467245), and Ambion RNase I (#AM2294) were obtained from Thermo Fisher Scientific (MA, USA).

### 2.2 Cell Culture

The mouse colon adenocarcinoma cell line MC38, its fluorescent/luciferase‐tagged derivatives (MC38‐GFP and MC38‐LUC), the mouse melanoma cell line B16‐F10, the firefly luciferase‐labeled mouse ovarian cancer cell line ID8‐LUC, and the HEK293T cell line were maintained in our laboratory [17, 18]. All lines were authenticated and tested negative for mycoplasma. The above cell lines and primary peritoneal macrophages were grown in high‐glucose DMEM (#C3113, VivaCell) with 10% FBS (#C04001, VivaCell) and 1% antibiotics (#P1400, Solarbio). Bone marrow‐derived macrophages (BMDMs) were differentiated and maintained in α‐MEM (#SH30265.01, HyClone, Utah, USA) containing 10% FBS (#C04001, VivaCell), antibiotics, and 40 ng/mL recombinant M‐CSF. For CD8^+^ T cell culture, complete RPMI‑1640 medium (#C11875500BT, Gibco, NY, USA) was prepared with 10% FBS (#Z7010FBS, Zeta Life, CA, USA), 1% penicillin‑streptomycin, 55 µM 2‑mercaptoethanol (#21985023, Gibco), 1% HEPES (#C0215, Beyotime), 1% L‑glutamine (#25030081, Thermo Fisher Scientific, MA, USA), 1% sodium pyruvate (#11360070, Thermo Fisher Scientific) and 1% MEM non‑essential amino acids (#11140050, Thermo Fisher Scientific). All cultures were incubated at 37 °C under 5% CO_2_ and saturated humidity.

### 2.3 Preparation of Tumor Cell‐Conditioned Medium (CM)

Conditioned medium (CM) was harvested from MC38 cells to simulate the soluble factors present in the tumor microenvironment in vitro. MC38 cells were grown to 80% confluence, then switched to DMEM with 1% FBS (10 mL per T‑75 flask). After 48 h of incubation, the supernatant was collected. This MC38‑primed medium was mixed 1:1 (v/v) with fresh complete medium to generate the final MC38‐CM, which was used for subsequent macrophage stimulation experiments.

### 2.4 Mice

We conducted all animal studies in compliance with the guidelines of the Institutional Animal Care and Use Committee of the Third Military Medical University (TMMU, Chongqing, China) (license SYXK(渝)2022‐0018) or Jinfeng Laboratory (Chongqing, China) (license SYXK(渝)2022‐0019). Mice were maintained under SPF conditions (12‑h light/dark cycle) and had free access to standard rodent chow and water. C57BL/6 wild‑type mice were purchased from GemPharmatech (Jiangsu, China). A C57BL/6J mouse line carrying a loxP‑flanked (fl) allele of *Pabpc4* (*Pabpc4* ^fl/−^) was obtained from Cyagen Biosciences (Suzhou, Jiangsu, China). Myeloid‑specific *Pabpc4* conditional knockouts were produced by mating *Pabpc4* ^fl/fl^ mice to *Lyz2*‑Cre mice (Cyagen Biosciences), employing a three‑step backcrossing scheme to generate *Pabpc4* ^fl/fl^; *Lyz2*‑Cre progeny (C4cKO). ‌For all in vivo experiments, animals were randomized to treatment groups via a computer‐generated code after genotyping. Littermates of the same sex and age (±1 week) were used to minimize genetic and environmental variability.

### 2.5 AAV‑Mediated Rescue of Spp1 Expression In Vivo

Recombinant Cre‐inducible adeno‑associated virus overexpressing mouse *Spp1* (AAV‑Spp1; #K25K1107, GV506 vector) and the corresponding empty‑vector control (AAV‑Vector; #CON314) were purchased from GeneChem (Shanghai, China). For in vivo rescue assays, mice were injected intravenously with 1 × 10^12^ viral genome particles of either AAV‑Spp1 or AAV‑Vector. Tumor implantation was initiated 30 days after viral administration to allow for stable transgene expression.

### 2.6 AOM/DSS‐Driven Colitis‐Associated CRC Model

Age‐matched wild‑type C57BL/6 mice (8–12 weeks) were subjected to the colitis‐associated colorectal tumorigenesis protocol. Mice were given an intraperitoneal AOM (10 mg/kg) injection first. Five days later, colorectal tumorigenesis was promoted via three cycles of DSS (2.5% w/v in drinking water; 5 days on, 14 days off). Mouse body weight was monitored every two days throughout the regimen. Animals were kept on regular chow and water after the final DSS cycle and euthanized on day 92 post‑AOM for colon harvest.

### 2.7 Mouse Models of Subcutaneous Tumor

Ctrl (*Pabpc4* ^fl/fl^) and C4cKO (*Pabpc4* ^fl/fl^; *Lyz2*‑Cre) mice (6 weeks old) received a subcutaneous injection in the groin region of 2 × 10^6^ MC38 or B16‑F10 cells suspended in 100 µL PBS. Tumors were allowed to grow for approximately 12 days, after which the animals were sacrificed, and the subcutaneous masses were dissected, imaged, and weighed.

### 2.8 Macrophage Adoptive Therapy Model of Subcutaneous Carcinomatosis

Lentiviral vectors for *Spp1* overexpression (OE) and negative control (NC), both carrying puromycin resistance, were obtained from GeneChem (Shanghai, China). Peritoneal macrophages (PMs) were isolated from Ctrl (*Pabpc4* ^fl/fl^) and C4cKO (*Pabpc4* ^fl/fl^; *Lyz2*‑Cre) mice and then transduced with the respective lentiviruses as recommended by the supplier, followed by puromycin selection to establish stable populations. Subsequently, 5 × 10^5^ transduced macrophages were mixed with 2 × 10^6^ MC38 or B16‐F10 cells in 100 µL of sterile PBS and co‐injected intraperitoneally into wild‐type C57BL/6 recipients. Approximately 12 days later, animals were sacrificed, and peritoneal tumor lesions were carefully excised, photographed, and quantified by weight.

### 2.9 Mouse Models of Peritoneal Carcinomatosis

#### 2.9.1 MC38 Colorectal Cancer Peritoneal Model

Ctrl (*Pabpc4* ^fl/fl^) and C4cKO (*Pabpc4* ^fl/fl^; *Lyz2*‑Cre) mice (6 weeks) received an intraperitoneal injection of 2 × 10^6^ MC38‑LUC cells in 100 µL PBS. Approximately 12 days later, D‑luciferin (150 mg/kg, i.p.) was administered, and bioluminescence was quantified using an IVIS Spectrum BL imaging system (PerkinElmer) once the signal reached a stable plateau. Tumors were then collected, photographed, weighed, and processed for flow cytometry.

#### 2.9.2 ID8 Ovarian Cancer Peritoneal Model

Female Ctrl and C4cKO mice (6 weeks) were similarly injected with 2 × 10^6^ ID8‑LUC cells. Approximately 40 days post‑injection, bioluminescence imaging was performed as described above. Mice were either euthanized for tumor imaging or maintained for survival analysis.

#### 2.9.3 Macrophage Adoptive Therapy Model of Peritoneal Carcinomatosis

To assess the therapeutic potential of macrophage‑based intervention, an adoptive co‑injection model was established. PMs and BMDMs were harvested from Ctrl (*Pabpc4* ^fl/fl^) and C4cKO (*Pabpc4* ^fl/fl^; *Lyz2*‑Cre) mice. For standard adoptive transfer, a mixture of 5 × 10^5^ macrophages and 2 × 10^6^ MC38 cells in 100 µL of sterile PBS was co‑injected i.p. into wild‑type C57BL/6 recipients (6 weeks). For SPP1 overexpression studies, 5 × 10^5^ macrophages transduced with lentivirus overexpressing *Spp1* (OE) or a negative control vector (NC) were mixed with 2 × 10^6^ MC38 or ID8‐LUC cells and administered i.p. in the same volume. Mice bearing MC38 peritoneal tumors were euthanized approximately 12 days post‐injection, while those bearing ID8 tumors were euthanized approximately 40 days post‐injection. Peritoneal tumor nodules were carefully excised, photographed, and weighed for subsequent analysis.

### 2.10 Mouse Models of Hepatic Metastasis Tumor

Liver metastasis was established by intrasplenic injection of MC38 cells. Under aseptic conditions, a left subcostal laparotomy incision exposed the spleen. A syringe needle was inserted superficially (∼0.5 mm deep) into the lower pole of the spleen and advanced parallel to the organ surface within the subcapsular plane. Then, 5 × 10^5^ MC38 cells in 50 µL PBS were slowly injected; successful delivery was confirmed by transient blanching and swelling of the splenic tissue. The needle was withdrawn, and the puncture site was immediately compressed with an alcohol‑saturated cotton tip for 2 min to minimize tumor cell leakage and provide local disinfection. After ensuring hemostasis, the spleen was repositioned and the abdominal wall was sutured in layers. Approximately 14 days post‑injection, mice were sacrificed. Livers were harvested, and metastatic burden was assessed by gross imaging and histopathological examination (hematoxylin and eosin staining).

### 2.11 Isolation of Peritoneal Macrophages (PMs)

We isolated primary peritoneal macrophages following a previously established protocol [17, 18]. Mice received an intraperitoneal injection of 3 mL sterile 3% thioglycolate broth to elicit an inflammatory response. After 72 h, mice were euthanized, and the abdominal skin was disinfected. Peritoneal cells were collected by lavage with chilled PBS. The resulting cell suspension was centrifuged, and the pellet was subjected to erythrocyte lysis (2 min, room temperature) using RBC lysis buffer (#R1010, Solarbio), and washed again with PBS. Cells were then resuspended in complete DMEM and plated. After 2 h of incubation at 37 °C, non‑adherent cells were rinsed away, leaving an adherent macrophage‐rich population for further use.

### 2.12 Isolation and Induction of Bone Marrow‐Derived Macrophages (BMDSs)

We generated BMDMs according to established protocols [17, 18]. Briefly, animals were sacrificed and surface‑disinfected. Marrow cells from tibiae/femurs were flushed with cold PBS and subjected to erythrocyte lysis (2 min, room temperature) using RBC lysis buffer, followed by another PBS rinse. After centrifugation, cells were resuspended in α‑MEM containing 10% FBS, antibiotics, and recombinant mouse M‑CSF (40 ng/mL), and plated in culture dishes. On days 3 and 5, culture medium was half‐replaced with fresh differentiation medium. On day 7, adherent mature BMDMs were harvested for subsequent experiments.

### 2.13 Quantitative Real‐Time PCR (qPCR)

Total cellular RNA was isolated from cells via an RNA isolation kit (#LS1040, Promega, WI, USA). cDNA was synthesized from 1 µg RNA using RT Master Mix (#RR036, TaKaRa, Japan). Quantitative PCR was conducted with qPCR mix (#RR820a, TaKaRa) through a CFX Connect instrument (Bio‑Rad, RRID: SCR_026760). Triplicate 20 µL reactions were analyzed. Target gene expression was normalized to β‑actin and quantified using the 2^−ΔΔCT^ method. Primers are detailed in Table S1.

### 2.14 mRNA Stability Assay

To assess mRNA stability, peritoneal macrophages were exposed to actinomycin D (10 µg/mL) to block de novo transcription. Cells were collected at designated intervals (0 h, 2 h, 4 h, 8 h, 12 h, 16 h, 20 h, and 24 h) for RNA extraction. *Spp1* mRNA levels were subsequently quantified by reverse transcription followed by qPCR. Subsequently, mRNA decay kinetics were analyzed, and the half‐life (t_1/2_) was calculated relative to the untreated (0 h) control.

### 2.15 RNA Immunoprecipitation (RIP) Assay

RIP was carried out using the RNA Immunoprecipitation Kit (#P0101, Geneseed, Guangzhou, China) to assess PABPC4‐*SPP1* mRNA interaction in macrophages. Following the supplier's protocol, approximately 1 × 10^7^ primary peritoneal macrophages were lysed in RIP‐lysis buffer containing protease and RNase inhibitors. After centrifugation (10,000 × *g*, 10 min, 4 °C), the supernatant was collected as whole‐cell extract. Two aliquots of the extract were reserved as Input controls for subsequent RNA and protein analysis. Protein A/G beads were pre‐coupled with 5 µg anti‐PABPC4 (#A5948; RRID: AB_2766680, Abclonal) or an equivalent amount of normal rabbit IgG (supplied with the kit) for 2 h at 4 °C, then mixed with the extract overnight at 4 °C with gentle rotation. Beads were magnetically recovered, washed extensively, and divided: approximately 10% of the sample was reserved for protein elution and the remainder for RNA extraction, both performed according to the kit instructions. The immunoprecipitated RNA was reverse‐transcribed and subjected to qPCR. Enrichment of *Spp1* mRNA was calculated by the 2^−ΔΔCT^ method, where ΔΔCT = (CT_IP_—CT_Input_)—(CT_IgG_—CT_Input_), and expressed as fold enrichment relative to the IgG control. The reserved protein fraction was analyzed by western blot to confirm successful immunoprecipitation of PABPC4.

### 2.16 Crosslinking‐Immunoprecipitation and qPCR (CLIP‐qPCR) Assay

For CLIP‐qPCR, peritoneal macrophages (1 × 10^7^ cells) were plated in 10‐cm dishes, washed with ice‐cold PBS, and covered with 6 mL of ice‐cold PBS before UV irradiation (254 nm, 150 mJ/cm^2^) in a UVP Crosslinker (CL‐3000, Analytik Jena UVP, USA) to induce covalent crosslinking between RNA‐binding proteins and directly contacted RNA bases. Subsequently, the target protein‐RNA complexes were immunoprecipitated and subjected to nuclease digestion. Cells were lysed in RIP buffer from RNA Immunoprecipitation Kit (#P0101, Geneseed) without adding RNase inhibitors. RNase I stock solution (Ambion RNase I, #AM2294, Thermo Fisher Scientific) was diluted with lysis buffer to two working concentrations: high (1:50) and low (1:500). Then, 10 µL of the diluted RNase I solution was added to 1 mL cell lysate for 3 min at 37 °C for 3 min to achieve either complete or partial RNA fragmentation. Digestion was terminated on ice, and lysates were cleared (12,000 × *g*, 10 min, 4 °C). Subsequent immunoprecipitation, RNA extraction, and qPCR were performed following the standard RIP protocol.

### 2.17 Dual‐Luciferase Reporter Assay

To further map PABPC4 binding on *SPP1* 3′UTR, endotoxin‐free plasmids were obtained from Tsingke Biotechnology (Beijing, China), including a *Pabpc4* expression plasmid and an empty control vector (both based on pcDNA3.1), as well as wild‐type (3′UTR‐wt) and mutant (3′UTR‐mt) *Spp1* reporter plasmids, an empty pmirGLO vector, and a PRL‐TK internal control plasmid. HEK293T cells were co‐transfected with the expression plasmid, reporter plasmid, and internal control via Lipofectamine 3000 (#L3000015, Thermo Fisher Scientific), with corresponding control groups arranged. After 48 h, luciferase signals were assayed with Dual‐Luciferase Kit (#E1901, Promega, USA) on a SpectraMax iD5 microplate reader (Molecular Devices, CA, USA).

### 2.18 Western Blotting

Macrophages were fully lysed, subjected to quantification and thermal denaturation, then resolved via 10% SDS‑PAGE. Subsequently, proteins were transferred onto PVDF membranes and blocked in 5% milk/TBST. Then, membranes were probed with primary antibodies: anti‐PABPC4 (#A5948; RRID: AB_2766680, Abclonal), anti‐SPP1 (#A21084, Abclonal), anti‐STAT1 (#9172; RRID: AB_2198300, Cell Signaling Technology), anti‐p‐STAT1 (Y701) (#AP0135; RRID: AB_2771563, Abclonal), anti‐p‐STAT1 (S727) (#AP1000; RRID: AB_2863892, Abclonal), anti‐p‐MLKL (S345) (#AP1550, Abclonal), anti‐p‐RIPK1 (S166) (#53286; RRID: AB_2925183, Cell Signaling Technology), anti‐p‐RIPK3 (S232) (#87148‐1‐RR; RRID: AB_3745370, Proteintech), and anti‐β‐actin (#66009‐1‐Ig; RRID: AB_2687938, Proteintech). Following TBST washes, membranes were treated with HRP‐conjugated secondaries. Signals were developed with ECL (#KF003, Affinity Biosciences) and recorded on the FUSION FX6 EDGE system (VILBER).

### 2.19 Glucose Content Detection

Glucose levels were measured with a commercial assay kit (#F006‐1‐1, Nanjing Jiancheng, China) based on the glucose oxidase method. Peritoneal macrophages (5 × 10^6^) were collected, washed, and resuspended in PBS. Cells were disrupted by ultrasonication (ice bath, 200 W, 5 s/cycle, 15 s intervals, repeated 5 cycles) and centrifuged (10,000 × *g*, 10 min, 4 °C). The clear supernatant was reacted with the kit working solution (37 °C, 10 min). Absorbance (505 nm) was recorded with a SpectraMax iD5 reader (Molecular Devices). Glucose content was determined from the standard curve and expressed as mmol/10^4^ cells.

### 2.20 Lactate Content Detection

Lactate was assayed with an enzymatic kit (#A019‐2‐2, Nanjing Jiancheng, China). Peritoneal macrophages (5 × 10^6^) were collected, washed, resuspended in PBS, disrupted by ultrasonication (ice bath, 200 W, 5 s/cycle, 15 s intervals, repeated 5 cycles), and centrifuged (10,000 × *g*, 10 min, 4 °C). The clear supernatant was reacted with enzyme working solution (37 °C, 3 min), followed by the addition of chromogenic solution and an additional 5 min incubation. Absorbance (546 nm) was recorded with a SpectraMax iD5 reader. Lactate content was calculated based on the standard curve and expressed as mmol/10^4^ cells.

### 2.21 ATP Quantification Assay

Cellular ATP was assayed with a bioluminescent kit (#S0027, Beyotime). Peritoneal macrophages (2 × 10^6^ cells) were collected, and the culture supernatant was discarded. Cells were lysed in pre‐chilled ATP lysis buffer (200 µL per well), then centrifuged (12,000 × *g*, 5 min, 4 °C), and supernatants were collected. An ATP standard curve was generated by serial dilution of ATP standards (0.01–10 µM) with ATP lysis buffer. In each detection well, 100 µL of ATP working solution was pre‐incubated for 5 min, then sample or standard was injected and mixed quickly. Signals were read immediately with a SpectraMax iD5 reader. ATP content was calculated based on the standard curve.

### 2.22 Immunofluorescence Staining

Colorectal tissues from AOM/DSS‐treated and control mice were harvested, gently rinsed with PBS, and prepared as “Swiss rolls” to preserve tissue architecture and enable longitudinal assessment. After fixation in 4% paraformaldehyde, tissues were embedded in paraffin and sectioned. Sections were deparaffinized, rehydrated, and subjected to antigen retrieval in citrate buffer (pH 6.0, #G1202, Servicebio, Wuhan, China) using a microwave heating method, followed by blocking (5% BSA/0.3% Triton X‑100, 1 h). For autofluorescence quenching, sections were treated with tissue autofluorescence quencher (#G1221, Servicebio). Primary anti‐PABPC4 (#A5948; RRID: AB_2766680, Abclonal) was applied overnight, followed by secondary antibody incubation. Subsequently, DAPI (#G1012, Servicebio) counterstaining was applied for 20 min. Whole‐section imaging was performed with a Pannoramic MIDI scanner (RRID: SCR_024834).

### 2.23 Hematoxylin and Eosin (H&E) Staining

Tissues were fixed, paraffin‐embedded, and sectioned (4 µm). Sections were deparaffinized, rehydrated, and stained with hematoxylin and eosin (#G1003, Servicebio) according to the manufacturer's protocol. After dehydration and clearing, slides were coverslipped with neutral balsam. Imaging was carried out using a bright‑field light microscope (NIKON ECLIPSE E100).

### 2.24 Immunohistochemistry (IHC) of human and Mouse Colorectal Tissues

Human CRC tissues from patients were obtained with approval from the Second Affiliated Hospital of Army Medical University (No. 2019‐研第103‐01). All samples were fixed prior to paraffin embedding. Mouse colorectum tissues from AOM/DSS‐treated and control mice were gently rinsed with PBS and prepared using the “Swiss‐roll” technique to preserve mucosal architecture for longitudinal analysis prior to fixation and paraffin embedding. Then, sections were cut, deparaffinized, rehydrated, and subjected to heat‐induced antigen retrieval in citrate buffer. Endogenous peroxidase was blocked (3% H_2_O_2_), and non‑specific binding was minimized with 5% BSA. Subsequently, sections were probed with primary antibodies targeting PABPC4 (#A5948; RRID: AB_2766680, Abclonal), CD68 (#GB113150; RRID: AB_2924885, Servicebio), and SPP1 (#GB11500; RRID: AB_3676309, Servicebio). After washing, slides were treated with HRP‑conjugated secondaries. Staining was developed with DAB, and sections were counterstained with hematoxylin, dehydrated, cleared, and coverslipped. Imaging was carried out with a bright‑field microscope (NIKON ECLIPSE E100).

### 2.25 In Vitro Phagocytosis Assay

To assess macrophage phagocytic capacity, peritoneal macrophages or bone marrow‑derived macrophages were co‑cultured with GFP‑expressing MC38 cells (1:4 ratio) for 4 h in 6‑well plates. Cells were then collected and subjected to flow cytometric staining. Macrophages were identified with PerCP/Cyanine5.5‑anti‑CD45 and APC/Cyanine7‑anti‑F4/80. Phagocytosis was quantified as the percentage of GFP^+^CD45^+^F4/80^+^ cells, representing macrophages that had internalized MC38‑GFP tumor cells. A complete list of antibodies is provided in Table S2.

### 2.26 Apoptosis Assay

Apoptosis was evaluated in primary PMs and BMDMs following 12 h stimulation with LPS (100 ng/mL), IL4 (20 ng/mL), or MC38‑conditioned medium (MC38‑CM). After treatment, both suspended and adherent cells were collected, pooled, washed, and stained with Annexin V‑APC/PI via a commercial kit (#E‐CK‐A217, Elabscience, Wuhan, China) per the supplier's instructions. Samples were then acquired on a CytoFLEX instrument (RRID: SCR_019627, Beckman Coulter). Apoptotic rates were determined as the sum of early (Annexin V^+^/PI^−^) and late (Annexin V^+^/PI^+^) fractions.

### 2.27 Reactive Oxygen Species (ROS) Level Detection

ROS was assayed with DCFH‐DA fluorescent probe (S0033, Beyotime). Peritoneal macrophages were collected, adjusted to 1 × 10^6^ cells/mL, and incubated with serum‐free DMEM containing 10 µM DCFH‐DA for 30 min. Cells were then washed, and fluorescence was measured via a CytoFLEX flow cytometer (RRID: SCR_019627, Beckman Coulter) at excitation/emission wavelengths of 488/525 nm, and ROS levels were quantified as mean fluorescence intensity (MFI).

### 2.28 In Vitro Co‐Culture Assay for Macrophage‑Mediated CD8^+^ T Cell Activation

CD8^+^ T cells were purified from mouse splenocytes via a negative selection kit (#480035, BioLegend). The isolated cells were seeded in anti‑CD3ε‐coated plates (5 µg/mL, 4 °C, 12 h) at a density of 1 × 10^5^ cells/cm^2^. Cells were activated and expanded for 48 h in complete RPMI‑1640 medium (formulation as described in the *Cell culture* section) supplemented with 1 µg/mL anti‑CD28 and 25 ng/mL IL‑2.

Primary peritoneal macrophages (PMs) were harvested from Ctrl (*Pabpc4* ^fl/fl^) and C4cKO (*Pabpc4* ^fl/fl^; *Lyz2*‑Cre) mice as described in the preceding section. PMs (3 × 10^5^/well) were seeded into U‑bottom 96‑well plates with complete DMEM. After 1 h of adherence at 37 °C, non‑adherent cells were discarded by gentle aspiration, and the adherent macrophage monolayer was washed once with pre‑warmed PBS.

Activated CD8^+^ T cells were harvested, washed, and resuspended in fresh RPMI‑1640, then added to the adherent PM monolayer at a 1:5 ratio (macrophage:CD8^+^ T cell). The co‑culture was maintained for 72 h at 37 °C in a 5% CO_2_ incubator. Following co‑culture, T cells were restimulated for 4 h with Cell Activation Cocktail to induce cytokine production, then harvested and stained with Zombie Green Fixable Viability Kit (#423111, BioLegend). Surface staining was performed using antibodies against CD45 (Alexa Fluor 700), CD3 (PE), and CD8α (PerCP/Cyanine5.5), followed by fixation/permeabilization with Foxp3 Buffer Set (#00‐5523‐00, eBioscience) and intracellular staining for IFNγ (APC), TNFα (Brilliant Violet 510), and Granzyme B (GzmB, Brilliant Violet 421). Samples were acquired on a BD LSRFortessa (RRID: SCR_018655, Becton, Dickinson and Company) and analyzed with FlowJo (v10.3.0; RRID: SCR_008520, TreeStar). The frequencies of IFNγ^+^, TNFα^+^, and GzmB^+^ cells were quantified within the live, singlet, CD3^+^CD8^+^ T cell population. A complete list of antibodies is provided in Table S2.

### 2.29 Preparing Single‐Cell Suspensions from Tumor Tissues, Spleens or Adipose Tissues

Single‑cell suspensions were generated from tumor, spleens, and visceral adipose tissues of euthanized mice for subsequent immune profiling by flow cytometry. All procedures were performed on ice or at 4 °C unless specified, using sterile, cold buffers to preserve cell viability.

#### 2.29.1 Peritoneal Tumor Tissue

Fresh peritoneal tumor tissues (∼1 g per sample) were minced into ∼1 mm^3^ fragments and enzymatically digested in RPMI‐1640 with collagenase IV (1 mg/mL), hyaluronidase (0.1 mg/mL), and DNase I (0.01 mg/mL) under gentle shaking (45‐60 min, 37 °C). The digestate was sequentially strained through 70‐µm cell strainers (#BS‐70‐CS, Biosharp), washed with cold PBS, and treated with RBC lysis buffer. The resulting pellet was suspended in FACS buffer.

#### 2.29.2 Subcutaneous Tumor Tissue

Subcutaneous tumors (∼1 g per sample) were processed similarly through mincing and enzymatic digestion. After filtration through 70‐µm strainers, cells were suspended in 40% Percoll (#17089109, Cytiva), then overlaid on a 70% Percoll cushion, and subjected to density gradient centrifugation (800 *g*, 20 min, room temperature). Interphase cells were collected, washed, RBC‐lysed, and finally resuspended in FACS buffer.

#### 2.29.3 Spleen

Spleens were placed on a 70‑µm strainer and gently dissociated with a syringe plunger. The strainer was rinsed with FACS buffer, then the cell suspension was collected. After RBC lysis, cells were washed and suspended in FACS buffer.

#### 2.29.4 Visceral Adipose Tissue

Adipose tissues were minced and digested in RPMI‐1640 containing collagenase IV (1 mg/mL) under gentle shaking (45 min, 37 °C), followed by filtration, centrifugation, RBC lysis, and final resuspension in FACS buffer.

### 2.30 Flow Cytometric Analysis of Immune Cell Infiltration

To characterize immune cell subsets within target tissues, single‑cell suspensions were first stained with viability dye (10 min), followed by surface marker staining with fluorochrome‑conjugated antibodies (45 min). Subsequently, cells were subjected to fixation/permeabilization with Foxp3 Buffer Set (#00‐5523‐00, eBioscience) after surface staining, followed by incubation with intracellular antibodies for 45 min. All antibodies are detailed in Table S2. After staining, cells were washed and strained via 40‑µm filters prior to acquisition. Samples were recorded by a BD LSRFortessa and analyzed with FlowJo. Live, single lymphocytes and myeloid cells were identified by forward/side scatter properties and viability dye exclusion. Immune subsets were defined as follows:

**T cells**: CD45^+^CD3^+^ cells. Subsets were further divided into CD4^+^ (CD45^+^CD3^+^CD4^+^CD8^−^) and CD8^+^ (CD45^+^CD3^+^CD4^−^CD8^+^) populations. The frequencies of IFNγ^+^, TNFα^+^, and GzmB^+^ cells were quantified within the respective T cell subsets.

**Macrophages**: CD45^+^F4/80^+^ cells after exclusion of CD90.2^+^ lymphocytes. Polarization states were defined as M1‑like (CD45^+^F4/80^+^CD11c^+^CD206^−^) and M2‑like (CD45^+^F4/80^+^CD11c^−^CD206^+^).

**Myeloid‑derived suppressor cells (MDSCs)**: CD11b^+^Gr1^+^ cells. Subsets were defined as polymorphonuclear MDSCs (PMN‐MDSCs: CD11b^+^Ly6G^+^Ly6C^low^) and monocytic MDSCs (M‑MDSCs: CD11b^+^Ly6G^−^Ly6C^high^).

### 2.31 RNA Sequencing and Transcriptomic Analysis

Primary peritoneal macrophages isolated from Ctrl (*Pabpc4* ^fl/fl^) and C4cKO (*Pabpc4* ^fl/fl^; *Lyz2*‑Cre) mice were treated with or without MC38‑CM for 24 h, constituting four experimental groups: Ctrl, C4cKO, Ctrl+CM, and C4cKO+CM, with three biological replicates per group. RNA was isolated with TRIzol (#R0016, Beyotime) and sequenced on the BGISEQ platform (BGI; contract No. F23A040008261_MOUeoziN). Reads were mapped to the mouse genome (GRCm38.p6, GCF_000001635.26). Gene‑level expression quantification was normalized as FPKM. Functional enrichment and expression heatmap of target genes were conducted using ClusterGVis (v0.99.9) in R (v4.4.1), with row‑scaled Z‑scores depicted in a blue‑red color scale (red for high expression, blue for low expression). Gene Set Enrichment Analysis (GSEA) was conducted with clusterProfiler (v4.8.3) on indicated gene sets from the Molecular Signatures Database (MSigDB). GSEA results were visualized using the GseaVis package (v0.0.5). All raw sequencing data produced in our study are publicly available in the Genome Sequence Archive (GSA, accession ID: CRA039011), hosted by the National Genomics Data Center, China National Center for Bioinformation, and are publicly accessible via https://ngdc.cncb.ac.cn/gsa.

### 2.32 Single‐Cell and Spatial Transcriptomics Analysis

Single‐cell RNA‐sequencing (scRNA‐seq) data of 20 clinical human CRC specimens across different stages were obtained from GSE178341 [19] (specific sample IDs are provided in Figure S2), and processed with Seurat (v5.1.0) in R software. Standard QC, normalization, and clustering (FindNeighbors/FindClusters, res  =  0.5) were applied, with Harmony (v1.2.1) used for batch correction. Cell identities were assigned by canonical markers [19, 20, 21, 22]. Expression patterns were visualized via DotPlot and VlnPlot functions in Seurat, integrated with ggplot2 (v3.5.2) and ggpubr (v0.6.0). Intercellular communication and ligand‐receptor interactions were explored with CellChat (v1.6.1) to compare communication probability across different disease stages.

To analyze the spatial expression distribution of the macrophage PABPC4 in CRC tissues with the spatial transcriptome dataset GSE225857 [23], Seurat was used with SCTransform normalization, PCA reduction, and spot clustering. The spatial distribution of gene expression was visualized using SpatialFeaturePlot. To specifically evaluate PABPC4 expression in macrophages within the spatial context, a macrophage‑enriched score was derived from weighted co‑expression of *CD68* and *PABPC4* per spot.

### 2.33 Bioinformatics Analysis of Clinical Data

Clinical RNA‑sequencing data for colon adenocarcinoma (COAD) were retrieved from the TCGA database. The relative abundance of immune cell populations within COAD tumors was deconvoluted using the xCell algorithm implemented in the immunedeconv package (v2.1.0), based on the expression levels of CD68, PABPC4, and SPP1. To evaluate the activity of the macrophage PABPC4‑SPP1 axis, a TAM_PABPC4 axis signature score was derived as the average z‑score of *CD68*, *LYVE1*, *PABPC4*, and *SPP1*. Similarly, a composite immune checkpoint signature score was computed based on the expression of *CTLA4*, *CD274* (PD‑L1), *PDCD1* (PD‑1), *PDCD1LG2 (*PD‑L2), *LAG3*, and *TIGIT*. All signature scores were derived using the calculate_sig_score function from the IOBR package (v0.99.0). The potential for tumor immune evasion was assessed with the TIDE algorithm (http://tide.dfci.harvard.edu/), along with the correlation between these two signature activities, which was statistically analyzed using the Hmsic package (v5.1‐3). Immunotherapy response was predicted from pre‑computed immunophenoscore (IPS) data available in the TCIA database (https://tcia.at/home). The IPS for patients who underwent anti‑PD‑1/CTLA‑4 immunotherapy was retrieved, and their correlation with the TAM_PABPC4 axis activity was assessed. Moreover, patients were stratified by median TAM_PABPC4 axis signature score, and overall survival differences were compared with the Mantel‑Cox log‑rank test via the survival package (v3.6‑4). Kaplan‑Meier curves were plotted with survminer (v0.4.9).

### 2.34 Statistical Analysis

Statistical tests were conducted with GraphPad Prism 9. Tumor weights for the two groups are shown as the mean ± SEM, while other data are presented as the mean ± SD. Two‐group comparisons were assessed by unpaired two‐tailed Student's *t*‐test, except for clinical samples (paired *t*‐test). For multi‐group comparisons, one‐way or two‑way ANOVA was applied as appropriate (for single or two independent variables, e.g., genotype and treatment). For omics data, the Wilcoxon rank‐sum test was employed. Survival differences were evaluated by Gehan‐Breslow‐Wilcoxon or log‐rank tests. Experiments were independently repeated on three separate occasions with biological replicates. Mice were randomly assigned to experimental groups before interventions. Significance levels: ^*^ *p* < 0.05, ^**^ *p* < 0.01, ^***^ *p* < 0.001; n.s., not significant.

## 3 Results

### 3.1 PABPC4 Acts Primarily Within Macrophages to Promote CRC Progression

To assess the function of PABPs in tumors, we initially examined their abundance across human tumor cohorts from TCGA and CPTAC databases. Compared to normal tissues, most PABPs were upregulated in tumors, with PABPC1 and PABPC4 exhibiting the most pronounced increases (Figure 1A,B; Figure S1A–F). Prognostic analysis based on expression quartiles indicated that high expression of PABPC4, but not other PABPs, was consistently associated with poor survival of patients with CRC (Figure 1C; Figure S1G–J). We further confirmed the marked upregulation of PABPC4 in an AOM/DSS‐induced murine model of spontaneous colorectal tumorigenesis (Figure 1D). These data indicate that PABPC4 may be an oncogenic regulator of CRC progression.

**FIGURE 1:**
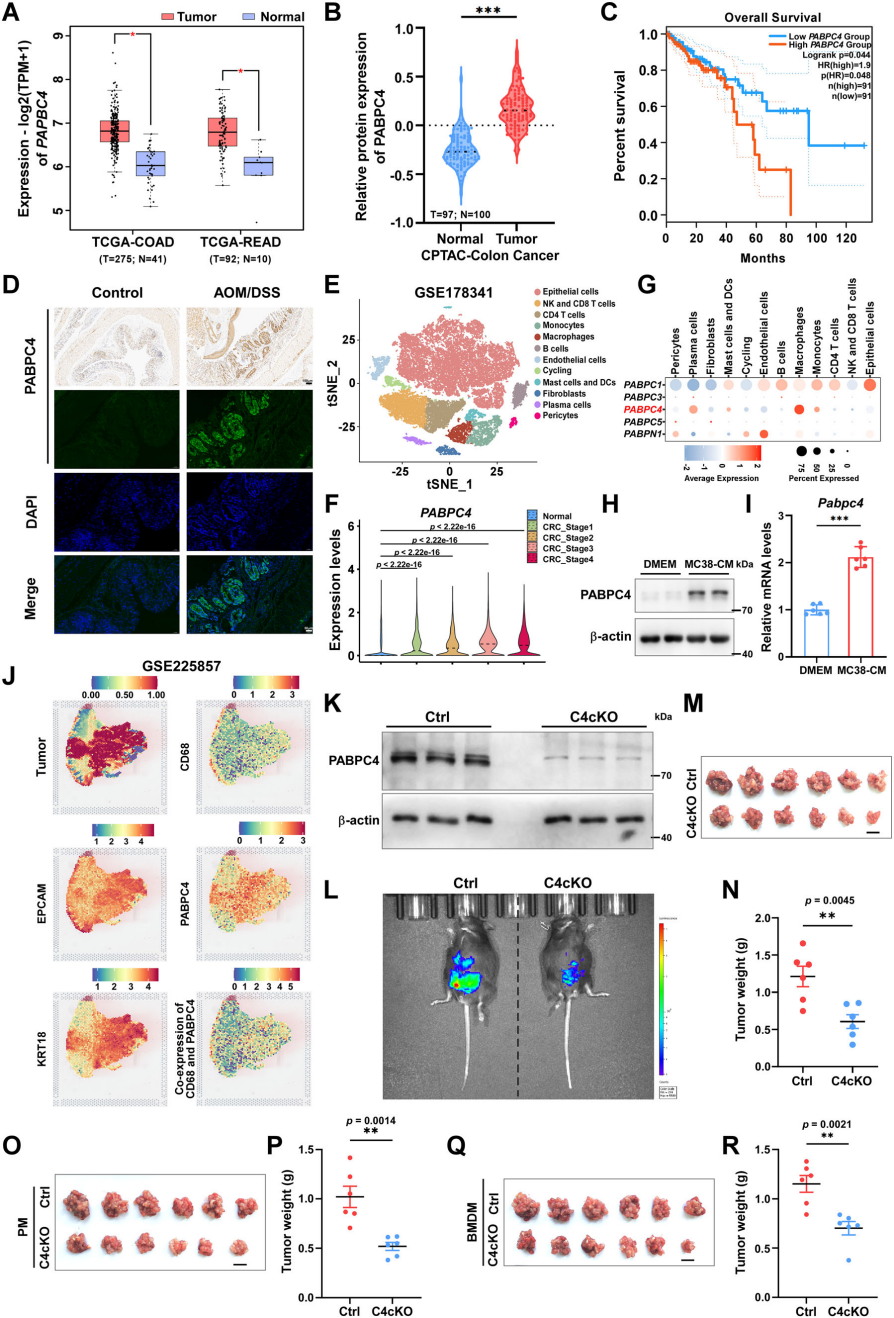
PABPC4 acts primarily within macrophages to promote CRC progression. (A) Differential mRNA expression of *PABPC4* in CRC versus normal tissues from TCGA‐COAD/READ cohorts, analyzed using the GEPIA2 online database. (B) Differential protein abundance of PABPC4 in CRC versus normal tissues, analyzed using the CPTAC colon cancer dataset. (C) Kaplan‐Meier survival curves for COAD and READ patients, stratified by high versus low quartile expression of *PABPC4* in the TCGA cohort (*n* = 91 per group). (D) Representative immunohistochemistry and immunofluorescence staining of PABPC4 in colons from AOM/DSS‑induced CRC mice and control mice. Scale bar, 50 µm. (E) t‑SNE visualization of major cell clusters identified by integrated single‑cell RNA‑seq analysis of 20 CRC specimens across different stages (GSE178341). (F) Violin plot showing normalized *PABPC4* expression levels across major cell clusters at different CRC stages. (G) Dot plot depicting PABPs expression across major clusters. (H) Western blotting detection of PABPC4 in PMs induced with or without conditioned medium (CM) of MC38 cells for 24 h. (I) qPCR analysis of *Pabpc4* mRNA levels in PMs treated as in (H) (*n* = 6 per group). (J) Spatial transcriptomic mapping of GSE225857 showing co‑expression of *PABPC4* and macrophage marker *CD68* in tumor versus adjacent non‑tumor regions of human CRC. (K) Western blotting confirmation of *Pabpc4* knockout in PMs isolated from *Pabpc4* conditional knockout (*Pabpc4* ^fl/fl^; *Lyz2*‐Cre, hereafter referred to as C4cKO) mice. (L) Representative bioluminescence images of mice bearing intraperitoneal MC38‑LUC tumors, demonstrating that macrophage *Pabpc4* knockout inhibits peritoneal metastasis. (M, N) Representative images (M) and quantified tumor weights (N) of peritoneal MC38‑LUC tumors harvested from C4cKO and control mice (*Pabpc4* ^fl/fl^, Ctrl) (*n* = 6 per group). Scale bar, 1 cm. (O, P) Representative images (O) and tumor weights (P) of peritoneal tumors from an adoptive macrophage transfer model, in which wild‑type C57BL/6 mice were co‑inoculated with MC38 cells and PMs derived from Ctrl or C4cKO mice (*n* = 6 per group). Scale bar, 1 cm. (Q, R) Representative images (Q) and tumor weights (R) of peritoneal tumors from an adoptive transfer model using BMDMs from Ctrl or C4cKO mice (n = 6 per group). Scale bar, 1 cm. Tumor weight data are presented as mean ± SEM, while other data are mean ± SD. Statistical analyses were performed with one‑way ANOVA (A), unpaired two‑tailed Student's *t*‑test (B, I, N, P, R), log‑rank test (C), or unpaired two‑sided Wilcoxon test (F). ^*^ *p* < 0.05, ^**^ *p* < 0.01, ^***^ *p* < 0.001.

Tumors comprise a complex ecosystem shaped by both malignant and stromal cells. To identify the cellular distribution of PABPC4 in CRC, we analyzed scRNA‐seq data from 20 clinical specimens across disease stages (GSE178341). After dimensionality reduction and clustering (Figure 1E; Figure S2A‐C), we demonstrated that PABPC4 expression increased with tumor stage (Figure 1F; Figure S2D) and was predominantly detected in macrophages (Figure 1G). Macrophage infiltration was elevated in tumors compared with normal tissues (Figure S2E), consistent with their established role in driving immunosuppression and disease progression. Re‐clustering of macrophages revealed that PABPC4 expression was obviously upregulated beginning at stage II (Figure S2F–K). In vitro, treatment of primary mouse peritoneal macrophages with CRC cell‐conditioned medium induced PABPC4 expression (Figures 1H,I). Spatial transcriptomics of clinical specimens from the GSE225857 dataset confirmed that PABPC4 was specifically overexpressed in macrophages within tumor regions (Figure 1J). These results suggest that PABPC4 is aberrantly upregulated in macrophages within the CRC microenvironment.

To investigate the biological significance of macrophage‐associated PABPC4, we generated genetic mouse models with conditional knockout of *Pabpc4* in macrophages (C4cKO) (Figure 1K) and tested them across multiple in vivo CRC models. We demonstrated that C4cKO notably attenuated subcutaneous tumor growth (Figure S2L,M) and intraperitoneal dissemination (Figure 1L–N), with no obvious effects on hepatic metastasis (Figure S2N,O). Furthermore, adoptive therapy with primary C4cKO macrophages also suppressed intraperitoneal dissemination of CRC cells (Figure 1O–R). These data indicated that PABPC4 acts primarily within macrophages to promote CRC progression. To further explore the potential broader relevance of macrophage PABPC4 beyond colorectal cancer, we extended our analysis to additional preclinical malignancy models. We found that C4cKO similarly restrained melanoma growth Figure S2P,Q) and peritoneal metastasis of ovarian cancer (Figure S2R,S), pointing to a possible tumor‐promoting function of macrophage PABPC4 that may not be restricted to a single cancer type.

### 3.2 Genetic Deletion of PABPC4 Attenuates the M2‐Like Activation of Macrophages

To investigate the mechanism through which macrophage PABPC4 enhances CRC progression, we first assessed the role of PABPC4 in macrophage function and survival. In vitro phagocytosis assays showed that PABPC4 deficiency did not alter macrophage phagocytic capacity (Figure 2A–D), with the key “eat me” and “don't eat me” signaling molecules not changed (Figure 2E). In contrast, cytokine profiling revealed that *Pabpc4* knockout significantly increased the expression of the proinflammatory cytokine *TNFα* (Figure 2F–H) while decreasing the anti‐inflammatory factors *Arg1* and *IL10* (Figure 2I–K). These results indicate that macrophage *Pabpc4* knockout restricts pro‐tumoral M2‐like activation.

**FIGURE 2:**
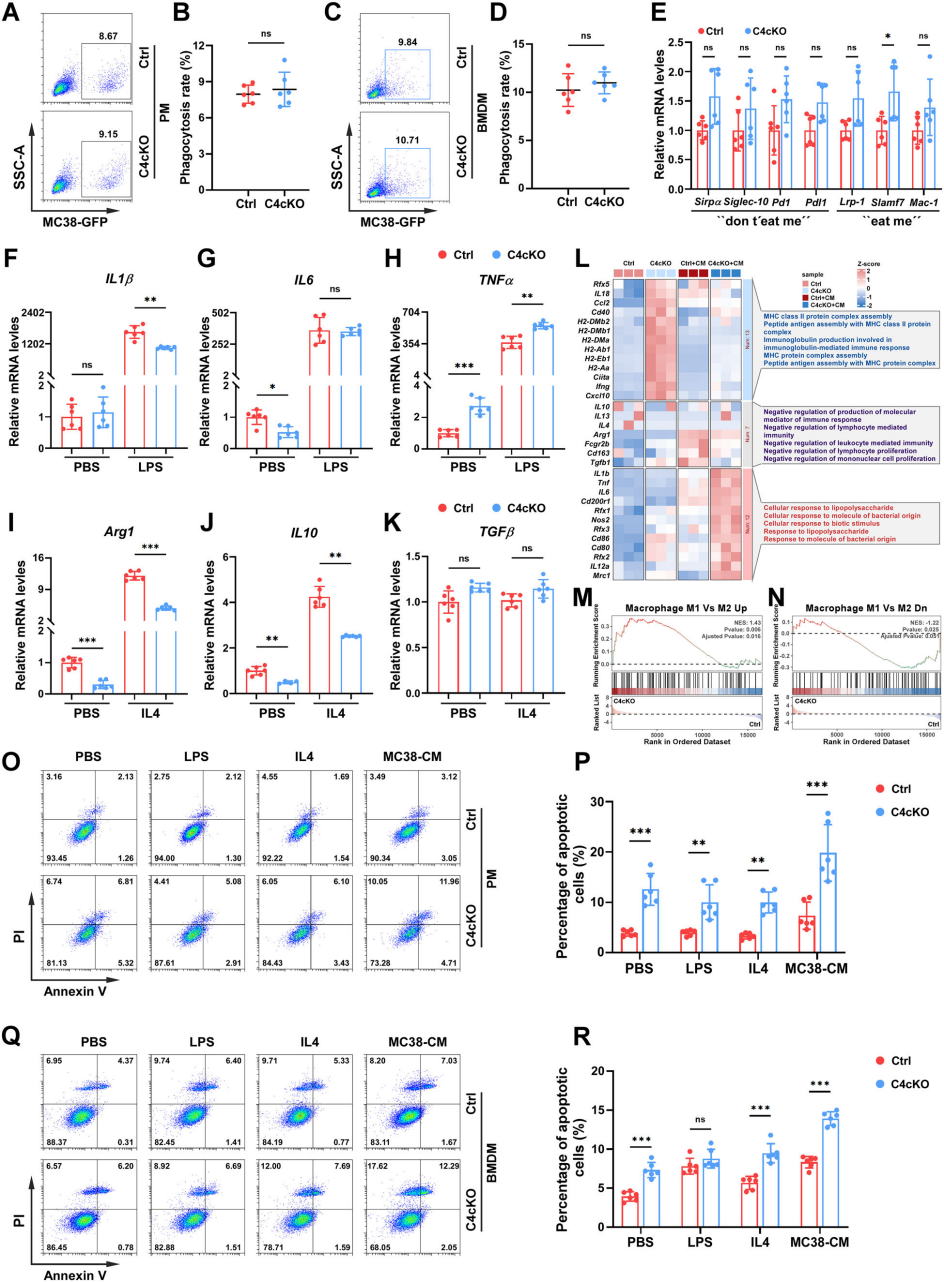
PABPC4 deficiency attenuates M2‐like polarization and promotes apoptosis in macrophages. (A–D) Representative flow cytometry plots and quantitative analysis of phagocytic uptake of GFP‑labeled MC38 (MC38‐GFP) cells by PMs (A, B) or BMDMs (C, D) isolated from control (*Pabpc4* ^fl/fl^, Ctrl) and C4cKO (*Pabpc4* ^fl/fl^; *Lyz2*‐Cre, C4cKO) mice (*n* = 6 per group). (E) qPCR analysis of phagocytosis‑associated genes (*Sirpα*, *Siglec‐10*, *Pd1*, *Pdl1*, *Lrp‐1*, *Slamf7* and *Mac‐1*) in Ctrl and C4cKO PMs (*n* = 6 per group). (F‐K) qPCR analysis of pro‑inflammatory (*IL1β*, *IL6*, *TNFα*; F‐H) and anti‑inflammatory (*Arg1*, *IL10*, *TGFβ*; I‐K) cytokine mRNA levels in Ctrl and C4cKO PMs treated with PBS, LPS (100 ng/mL), or IL4 (20 ng/mL) for 12 h (*n* = 6 per group). (L) RNA‐seq heatmap of M1‑/M2‑associated cytokine genes in Ctrl and C4cKO PMs, with or without 24 h of MC38‑CM treatment (*n* = 3 per group). (M, N) GSEA plots showing variation trend of the M1‐up (M) and M1‐down (N) signatures in C4cKO PMs relative to controls. (O‐R) Flow cytometry plots and quantitative analysis of apoptotic PMs (O, P) or BMDMs (Q, R) from Ctrl and C4cKO mice following 12 h treatment with PBS, LPS (100 ng/mL), IL4 (20 ng/mL), or MC38‑CM (*n* = 6 per group). Data are shown as mean ± SD. Statistical significance was assessed via unpaired two‑tailed Student's *t*‑test (B, D, E), or two‐way ANOVA (F‐K, P, R). ns stands for not significant, ^*^ *p* < 0.05, ^**^ *p* < 0.01, ^***^ *p* < 0.001.

We next performed transcriptomic sequencing of PABPC4‐deficient macrophages (Figure S2T). Heatmap analysis of cytokine‐associated genes showed that genes linked to M1‐like status were broadly upregulated, while those M2‐like genes were downregulated upon *Pabpc4* loss (Figure 2L). Consistently, GSEA using established M1 versus M2 macrophage signatures confirmed enrichment of the “COATES_MACROPHAGE_M1_VS_M2_UP” set and suppression of the “COATES_MACROPHAGE_M1_VS_M2_DN” set in *Pabpc4‐*knockout macrophages (Figure 2M,N). Additionally, apoptosis assays indicated that PABPC4 deletion promoted macrophage cell death, especially in the presence of CRC cell‐conditioned medium (Figure 2O–R), implying that PABPC4 loss may reduce the frequencies of TAMs within the TME. Taken together, these results demonstrate that PABPC4 deficiency could inhibit the M2‐like polarization of macrophages and enhance their susceptibility to apoptosis, thereby attenuating the protumoral role of TAMs in CRC.

### 3.3 Macrophage PABPC4 Deficiency Augments the Local and Systemic Antitumor Immunity in Mouse Tumor Models

Given that PABPC4 ablation inhibits M2‐like macrophage polarization, we next investigated its impact on the broader tumor immune landscape. Flow cytometric analysis of peritoneal tumors revealed that C4cKO reduced total macrophage infiltration while skewing the macrophage compartment toward an M1‐like rather than an M2‐like phenotype (Figure 3A–D; Figure S3A). Furthermore, C4cKO obviously diminished the frequency of myeloid‐derived suppressor cells (MDSCs), with a pronounced reduction in the polymorphonuclear subset (PMN‐MDSCs) (Figure 3E–H; Figure S3B). C4cKO increased the frequencies of tumor‐infiltrating T cells and CD8^+^ subset (Figure 3I–L; Figure S3C). Critically, the effector molecules IFNγ, TNFα, and granzyme B (GzmB) in T cells and the subsets were all largely induced in the TME of C4cKO mice (Figure 3M–R).

**FIGURE 3:**
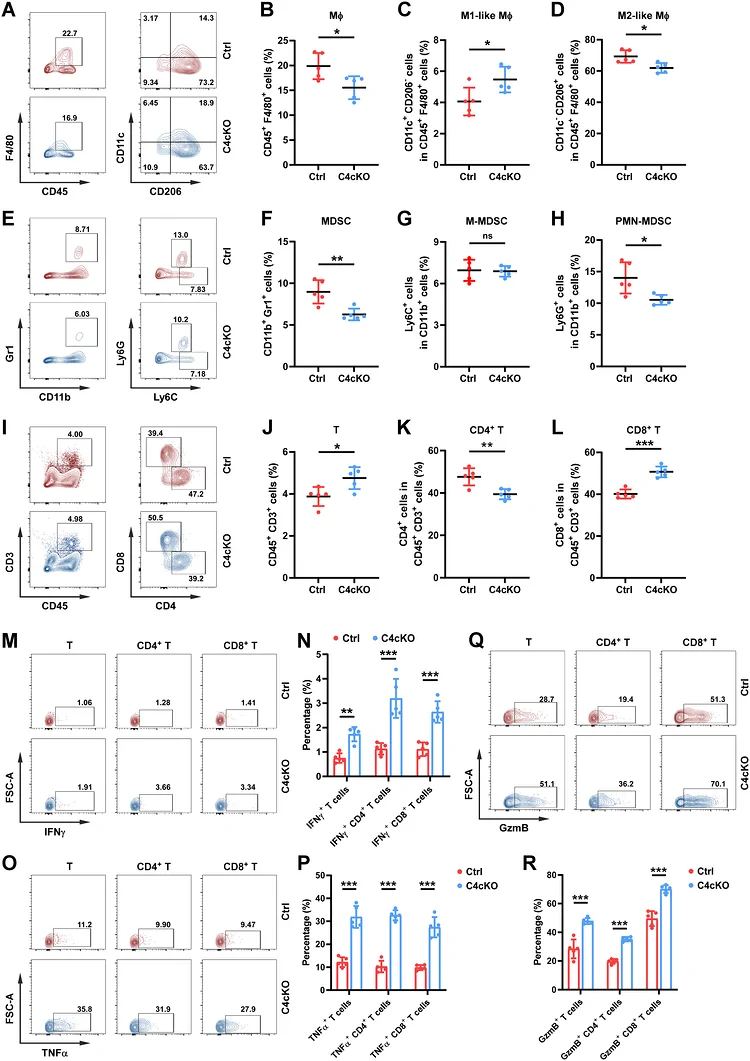
Macrophage PABPC4 ablation improves the immunosuppressive TME in CRC. (A–D) Flow cytometry analysis of macrophage subsets in peritoneal tumors from MC38‐bearing control (*Pabpc4* ^fl/fl^, Ctrl) and *Pabpc4* conditional knockout (*Pabpc4* ^fl/fl^; *Lyz2*‐Cre, C4cKO) mice (A). Quantitative analysis of total macrophages (B), M1‑like (CD11c^+^CD206^−^, C), and M2‑like (CD11c^−^CD206^+^, D) cells within CD45^+^F4/80^+^ populations (*n* = 5 per group). (E–H) Flow cytometry analysis of MDSC subsets in the same tumor models (E). Quantitative analysis of total MDSCs (CD45^+^CD11b^+^Gr‑1^+^, (F), M‐MDSCs (CD11b^+^Ly6G^−^Ly6C^high^, (G), and PMN‐MDSCs (CD11b^+^Ly6G^+^Ly6C^low^, (H) subsets (*n* = 5 per group). (I–L) Representative flow cytometry plots (I) and quantitative analysis of total T cells (CD45^+^CD3^+^, (J), CD4^+^ (CD3^+^CD4^+^CD8^+^, (K), and CD8^+^ (CD3^+^CD4^−^CD8^+^, (L) T cells in peritoneal tumors from Ctrl and C4cKO mice (*n* = 5 per group). (M–R) Frequencies of IFNγ^+^ (M, N), TNFα^+^ (O, P), and GzmB^+^ (Q, R) cells within total T cells, CD4^+^ and CD8^+^ T cell populations (*n* = 5 per group). Data are presented as mean ± SD. Statistical analyses were assessed with unpaired two‑tailed Student's *t*‑test (B–D, F–H, J–L, N, P, R). ns stands for not significant, ^*^ *p* < 0.05, ^**^ *p* < 0.01, ^***^ *p* < 0.001.

We extended TME analysis to the visceral adipose tissues, key sites of peritoneal metastasis of CRC at the early stage. The results showed that C4cKO reduced the frequencies of total macrophages, M2‐like macrophages (Figure S4A–D), and MDSCs (Figure S4E–H). Although the T cell frequencies were unchanged (Figure S4I–L), their effector function was notably enhanced by C4cKO, as indicated by elevated expression of IFNγ, TNFα, and GzmB (Figure S4M–R). In addition, systemic immune changes were assessed in the spleen. While splenic macrophage frequencies were unaltered (Figure S5A–D), MDSC levels were reduced by C4cKO (Figure S5E–H). Simultaneously, total T cell counts (Figure S5I–L) and their effector factors were amplified (Figure S5M–R) in C4cKO mice. Finally, in subcutaneous tumors, PABPC4 ablation confirmed a consistent pattern: decreased frequencies of M2‐like macrophages (Figure S6A–D) and MDSCs (Figure S6E–H), as well as increased infiltration of T cells (Figure S6I–L), IFNγ^+^ T cells, and the subsets (Figure S6M–P). Collectively, these results indicate that macrophage *Pabpc4* deletion enhances the local and systemic antitumor immunity in mouse tumor models.

### 3.4 Macrophage PABPC4 Drives CD8^+^ T Cell Dependent CRC Progression

To decipher the mechanism by which macrophage PABPC4 shapes the TME to promote CRC, we analyzed cell‐cell communication within our integrated multi‐stage single‐cell RNA‐seq datasets. Since advanced tumor stages are associated with metastasis, therapy resistance, and poor survival, we compared communication networks between late‐stage (stage IV) tumors and normal tissues. We observed a marked increase in signaling originating from macrophages in stage IV tumors, with enhanced communication to epithelial, NK, CD8^+^ T, CD4^+^ T, monocytic, and other macrophage populations (Figure 4A,B). The interaction between macrophages and the NK & CD8^+^ T cell cluster was among the most significantly strengthened in stage IV CRC (Figure 4A,B). Further sub‐clustering confirmed that the NK & CD8^+^ T cell cluster was predominantly composed of CD8^+^ T cells (Figure 4C–E). In line with this, gene‐set enrichment analysis of PABPC4‐deficient macrophages revealed significant upregulation of T cell activation and immune response signatures (Figure S7A,B). These results suggest that macrophages suppress immunity in CRC largely through modulation of T cell function.

**FIGURE 4:**
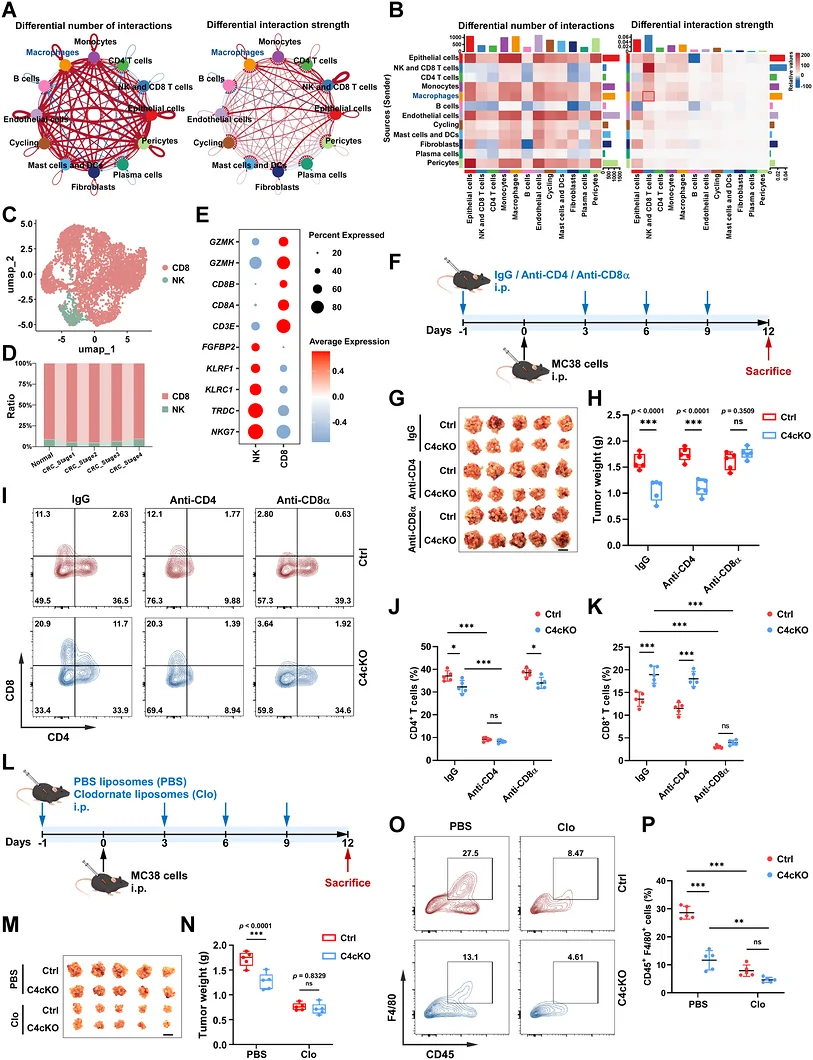
Macrophage PABPC4 drives CD8^+^ T cell‐dependent CRC progression. (A, B) Cell‐cell communication analysis of integrated scRNA‑seq cohort (linked to Figure 1). Circle plot (A) and heatmap (B) showing significantly altered interactions between macrophages and other cell subsets in stage IV CRC compared to normal tissues (red: increased; blue: decreased). (C) UMAP visualization of the NK and CD8^+^ T cell subclusters. (D) Bar plot of NK/CD8^+^ T cell proportions across CRC stages. (E) Dot plot of canonical NK/CD8^+^ T cell markers across subclusters. (F) Schematic illustration of the in vivo T cell depletion strategy. Control (*Pabpc4* ^fl/fl^, Ctrl) and *Pabpc4* conditional knockout (*Pabpc4* ^fl/fl^; *Lyz2*‐Cre, C4cKO) mice bearing intraperitoneal MC38 tumors were injected intraperitoneally with IgG isotype control, anti‐CD4 or anti‐CD8α neutralizing antibodies (100 µg per mouse per injection). (G, H) Representative images (G) and quantified tumor weights (H) of peritoneal MC38 tumors harvested from Ctrl and C4cKO mice following CD4^+^ or CD8^+^ T cell depletion (*n* = 5 per group). Scale bar, 1 cm. (I–K) Flow cytometry verification of T cell depletion. Representative plots (I) and quantitative analysis of CD4^+^ (J) and CD8^+^ (K) T cells in peritoneal tumors from the indicated groups (*n* = 5 per group). (L) Schematic illustration of the in vivo macrophage depletion strategy. Ctrl and C4cKO mice bearing intraperitoneal MC38 tumors were injected intraperitoneally with PBS liposomes (control, PBS) or clodronate liposomes (macrophage depletion, Clo) at a dose of 200 µL/mouse/injection. (M, N) Representative images (M) and quantified tumor weights (N) of peritoneal MC38 tumors harvested from Ctrl and C4cKO mice with or without macrophage depletion (*n* = 5 per group). Scale bar, 1 cm. (O, P) Flow cytometric confirmation of macrophage depletion. Representative plots (O) and quantitative analysis of total macrophages (CD45^+^F4/80^+^, P) in peritoneal tumors from the indicated groups (*n* = 5 per group). Data are shown as mean ± SD. Statistical analyses were assessed via two‑way ANOVA (H, J, K, N, P). ns stands for not significant, ^*^ *p* < 0.05, ^**^ *p* < 0.01, ^***^ *p* < 0.001.

We next directly investigated this macrophage‐T cell axis in vivo. In intraperitoneal xenograft tumor models, selective removal of CD8^+^ T cells (not CD4^+^ T cells) fully abrogated the C4cKO‐mediated tumor suppression (Figure 4F–H). The efficient depletion of the respective T cell subsets by blocking antibodies was confirmed by flow cytometry (Figure 4I–K). Consistently, systemic depletion of macrophages by clodronate liposomes also abolished the antitumor effect of C4cKO (Figure 4L–P), further indicating that macrophages serve as the key effector cells and their interaction with CD8^+^ T cells is essential for antitumor immunity.

To further explore the direct cross‐talk between macrophages and CD8^+^ T cells, we employed an in vitro coculture system. The results showed that CD8^+^ T cells cocultured with *Pabpc4*‐knockout macrophages exhibited significantly higher expression of the cytotoxic effector molecules IFNγ, TNFα, and GzmB compared to those cocultured with control macrophages (Figure 5A–F). We next determined whether macrophage depletion in vivo could block the immunostimulatory effects of C4cKO in MC38 tumor‐bearing mice. As expected, systemic depletion of macrophages by clodronate liposomes abolished C4cKO‐related alterations of M1/M2 frequencies in the TME (Figure 5G–I). Accordingly, macrophage clearance markedly diminished the enhancement of CD8^+^ T cell tumor infiltration (Figure 5J,K) and the upregulation of IFNγ, TNFα, and GzmB (Figure 5L–Q) observed in tumors from C4cKO mice. These results demonstrate that PABPC4 operates through macrophages to directly inhibit CD8^+^ T cell antitumor function, thereby facilitating CRC progression.

**FIGURE 5:**
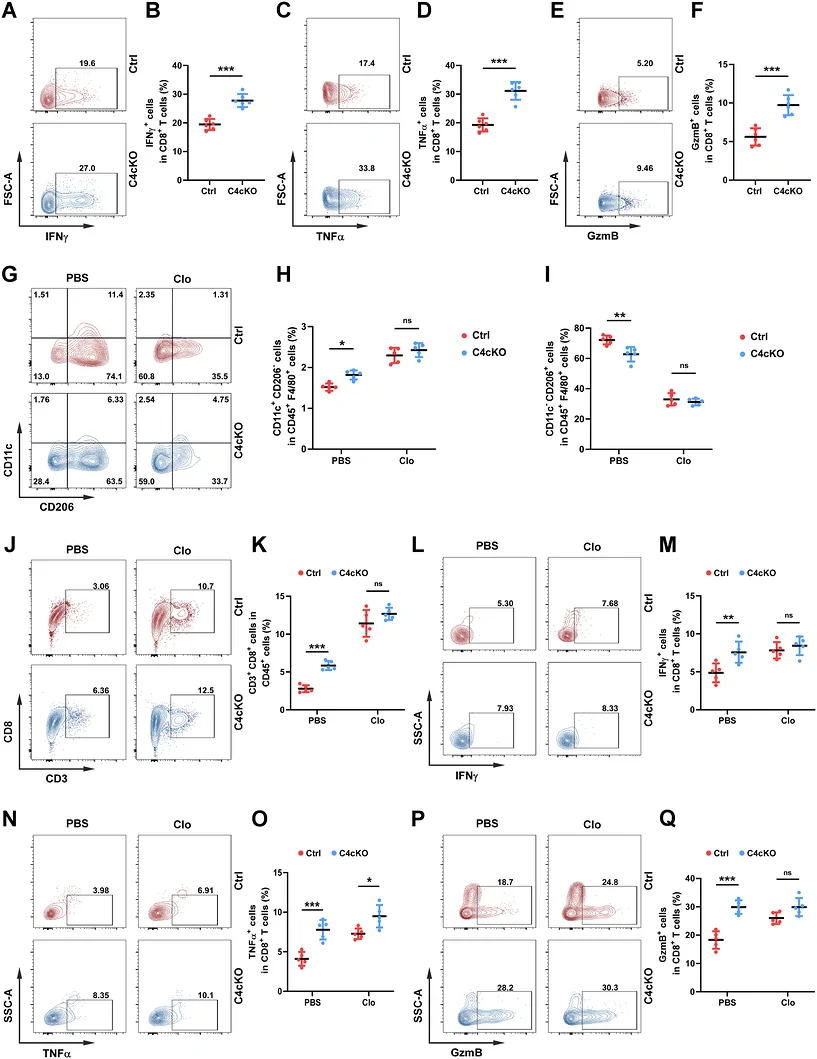
Macrophage PABPC4 ablation enhances CD8^+^ T cell activity in the TME. (A‐F) In vitro co‑culture assay. PMs isolated from control (*Pabpc4* ^fl/fl^, Ctrl) or *Pabpc4* conditional knockout (*Pabpc4* ^fl/fl^; *Lyz2*‐Cre, C4cKO) mice were co‑cultured with activated CD8^+^ T cells (1:5, 72 h). Representative plots and quantitative analysis of IFNγ^+^ (A, B), TNFα^+^ (C, D), and GzmB^+^ (E, F) cells within the CD8^+^ T cell population (*n* = 6 per group). (G–I) Flow cytometry plots (G) and quantitative analysis of M1‑like (CD11c^+^CD206^−^, H), and M2‑like (CD11c^−^CD206^+^, I) macrophages among CD45^+^F4/80^+^ cells in peritoneal tumors from MC38‐bearing Ctrl and C4cKO mice with clodronate liposomes (macrophage depletion, Clo) or PBS liposomes (control, PBS) (*n* = 5 per group). (J, K) Flow cytometry plots (J) and quantitative analysis of CD8^+^ T cells (K) in peritoneal tumors from the indicated groups (*n* = 5 per group). (L–Q) Frequencies of IFNγ^+^ (L, M), TNFα^+^ (N, O), and GzmB^+^ (P, Q) cells within CD8^+^ T cells in peritoneal tumors from Ctrl and C4cKO mice with or without macrophage depletion (n = 5 per group). Data are shown as mean ± SD. Statistical analyses were performed with unpaired two‑tailed Student's *t*‑test (B, D, F) or two‑way ANOVA (H, I, K, M, O, Q). ns stands for not significant, ^*^ *p* < 0.05, ^**^ *p* < 0.01, ^***^ *p* < 0.001.

### 3.5 Macrophage PABPC4‐SPP1 Axis Suppresses CD8^+^ T Cell Activity and CRC Progression

To identify the specific pathway through which PABPC4 impairs CD8^+^ T cell function in CRC, we first analyzed macrophage‐derived signaling pathways that were markedly upregulated in stage IV tumors versus normal tissues, along with significantly enhanced macrophage‐T cell ligand‐receptor pairs. This screen identified several pathways, including CSF3, IL4, SEMA7, SPP1, and TNF, that were strikingly induced in advanced diseases (Figure 6A; Figure S7C). Visualization of the corresponding macrophage‐expressed ligands in transcriptomic data from *Pabpc4*‐knockout macrophages revealed that Spp1 exhibited the most downregulation (Figure 6B), a finding corroborated by qPCR and western blotting (Figure 6C,D). Interaction hierarchy visualization and cellular role analysis further demonstrated that SPP1 signaling was indeed significantly heightened between macrophages and CD8^+^ T cells in CRC (Figure 6E; Figure S7D). Given that macrophage‐derived SPP1 is known to maintain M2‐like polarization and foster immunosuppression [24, 25], we speculated that *Pabpc4* knockout enhances antitumor immunity, at least in part, through inhibiting SPP1 expression in macrophages.

**FIGURE 6:**
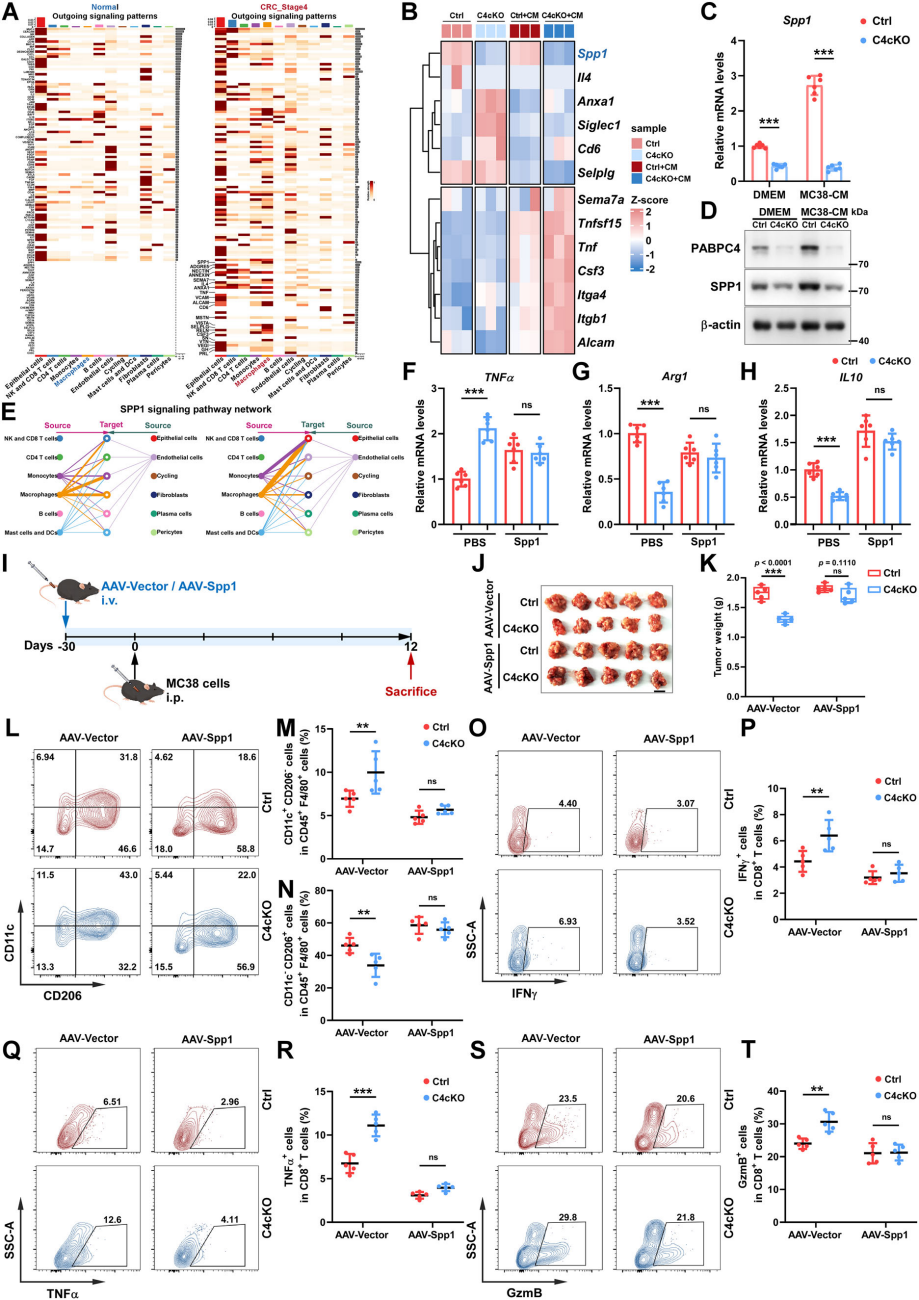
Macrophage PABPC4 deletion suppresses CRC progression by downregulating SPP1 signaling, thereby inhibiting M2 polarization and potentiating CD8^+^ T cell activity. (A) Heatmap showing significantly altered signaling pathways originating from macrophages in stage IV CRC compared to normal tissues. (B) Heatmap of macrophage‑derived ligands corresponding to the altered signaling pathways (identified in A) in Ctrl (*Pabpc4* ^fl/fl^) and C4cKO (*Pabpc4* ^fl/fl^; *Lyz2*‐Cre) PMs, derived from RNA‑seq data. (C) qPCR analysis of *Spp1* mRNA levels in Ctrl and C4cKO PMs after 24 h of MC38‐CM treatment versus untreated control. (n = 6 per group). (D) Western blotting of PABPC4 and SPP1 protein in Ctrl and C4cKO PMs following 24 h exposure to MC38‐CM or vehicle control. (E) Hierarchical visualization of cell‑cell communication networks mediated by the SPP1 signaling pathway in stage IV CRC. (F‐H) qPCR of *TNFα* (F), *Arg1* (G), and *IL10* (H) mRNA in Ctrl and C4cKO PMs upon recombinant Spp1 (100 ng/mL, 16 h) treatment versus PBS (*n* = 6 per group). (I) Schematic illustration of the in vivo Spp1 restoration strategy. Ctrl and C4cKO mice received a tail vein injection of AAV‐Vector or AAV‐Spp1 (1 × 10^12^ viral genome particles in 100 µL per mouse) 30 days prior to tumor inoculation. (J, K) Representative images (J) and quantified tumor weights (K) of peritoneal MC38 tumors harvested from Ctrl and C4cKO mice with or without Spp1 restoration (*n* = 5 per group). Scale bar, 1 cm. (L–N) Flow cytometry plots (L) and quantitative analysis of M1‑like (M) and M2‑like (N) macrophages in peritoneal tumors from the indicated groups (*n* = 5 per group). (O–T) Frequencies of IFNγ^+^ (O, P), TNFα^+^ (Q, R), and GzmB^+^ (S, T) cells within CD8^+^ T cells in peritoneal tumors from Ctrl and C4cKO mice with or without Spp1 restoration (*n* = 5 per group). Data are shown as mean ± SD. Statistical analyses were performed with two‑way ANOVA (C, F‐H, K, M, N, P, R, T). ns stands for not significant, ^**^ *p* < 0.01, ^***^ *p* < 0.001.

We next performed functional rescue experiments. In vitro, addition of recombinant SPP1 blocked the upregulation of TNFα and the downregulation of Arg1 and IL10 caused by *Pabpc4* knockout in macrophages (Figure 6F–H). More importantly, in vivo restoration of SPP1 via Cre‐inducible adeno‐associated virus (AAV)‐mediated overexpression largely abrogated the tumor‐suppressive effect of C4cKO in peritoneal metastasis (Figure 6I–K). Immune profiling of the TME confirmed that SPP1 replenishment blocked the C4cKO‐induced increase of M1‐like TAMs and decrease of M2‐like ones (Figure 6L–N; Figure S7E,F). Concurrently, C4cKO‐induced enhancement of CD8^+^ T cell infiltration and effector molecule production (IFNγ, TNFα, and GzmB) was also abolished by the supplementation of SPP1 (Figure 6O–T; Figure S7G,H). Moreover, in multiple adoptive transfer tumor models using macrophages transduced with lentivirus to overexpress SPP1, SPP1 overexpression significantly attenuated the anti‐tumor effect of macrophages derived from C4cKO mice (Figure S7I–O). Taken together, these results indicate that PABPC4 promotes CRC progression by upregulating SPP1 in macrophages, which in turn suppresses CD8^+^ T cell function.

To further clarify the receptor signaling pathway through which the PABPC4‐SPP1 axis regulates CD8^+^ T cells, we analyzed ligand‐receptor contributions within the SPP1 signaling network. This analysis identified the SPP1‐CD44 axis as the most significantly enriched contributor to the enhanced SPP1 signaling in CRC (Figure S8A). Given that CD44 is a well‐established receptor through which SPP1 acts on CD8^+^ T cells [26, 27], we hypothesized that the PABPC4‐SPP1 axis modulates CD8^+^ T cell anti‐tumor activity via CD44 signaling. To validate this, we blocked CD44 in macrophage‐T cell co‐cultures using a neutralizing antibody. This blockade significantly attenuated the upregulation of CD8^+^ T cell effector molecules (IFNγ, TNFα, and GzmB) and the downregulation of the immune checkpoint PD‐1 induced by C4cKO‐derived macrophages (Figure S8B–I). These findings indicate that the macrophage PABPC4‐SPP1 axis regulates CD8^+^ T cell anti‐tumor activity through CD44 receptor signaling.

Furthermore, given that SPP1 markedly diminished the reduction of immunosuppressive macrophages in the tumor microenvironment following *Pabpc4* knockout, we investigated whether SPP1 mediates the effect of *Pabpc4* deletion on macrophage susceptibility to cell death. To this end, we analyzed changes in gene sets related to cell death regulation in *Pabpc4*‐knockout macrophages. Although metabolic reprogramming is closely linked to cell death, *Pabpc4* deficiency did not induce significant alterations in metabolic pathways or related biochemical parameters (Figure S9A–E). In contrast, the necroptosis pathway was robustly activated (Figure S9A,F,G). STAT1, an upstream regulator of necroptosis known to be suppressed by SPP1 in macrophages [28], was upregulated and activated upon *Pabpc4* knockout (Figure S9F,G). Notably, SPP1 overexpression substantially attenuated the *Pabpc4* knockout‐induced upregulation of STAT1, the necroptosis markers p‐MLKL, p‐RIPK1, and p‐RIPK3, as well as the heightened sensitivity to cell death in macrophages exposed to CRC cell‐conditioned medium (Figure S9G,H). Collectively, genetic ablation of PABPC4 in macrophages disrupts this SPP1‐dependent immunosuppressive axis, leading to restored antitumor immunity and restricted tumor growth.

### 3.6 Elevated Activity of the PABPC4‐SPP1 Axis in Macrophages Is Correlated with Potential Immunotherapy Resistance and Unfavorable Prognosis in CRC Patients

Given the role of PABPC4 as a post‐transcriptional regulator, we examined whether it governs SPP1 expression by modulating mRNA stability. Measurement of *Spp1* mRNA decay kinetics revealed a notably shortened half‐life following *Pabpc4* knockout (Figure 7A,B). RIP assays verified direct binding of PABPC4 to *Spp1* mRNA in macrophages (Figure 7C), indicating that PABPC4 enhances *Spp1* expression by binding to and stabilizing its transcript. Subsequent CLIP‐qPCR analysis showed that under partial fragmentation (low RNase I), significant enrichment of the 3′UTR region was retained, whereas the CDS region was no longer enriched; under complete fragmentation (high RNase I), enrichment in both regions was abolished (Figure 7D). These results collectively indicate that the PABPC4‐binding region on *Spp1* mRNA resides within its 3′UTR. We then used the catRAPID omics v2 database (https://tools.tartaglialab.com/) to predict the specific binding site, generated wild‐type and mutant *Spp1* 3′UTR reporters, and co‐transfected them with a *Pabpc4* expression plasmid. Luciferase assays demonstrated that PABPC4 stabilized the wild‐type *Spp1* 3′UTR but had no stabilizing effect on the mutant 3′UTR, confirming this site as the interaction interface between PABPC4 and the *Spp1* 3′UTR (Figure 7E).

**FIGURE 7:**
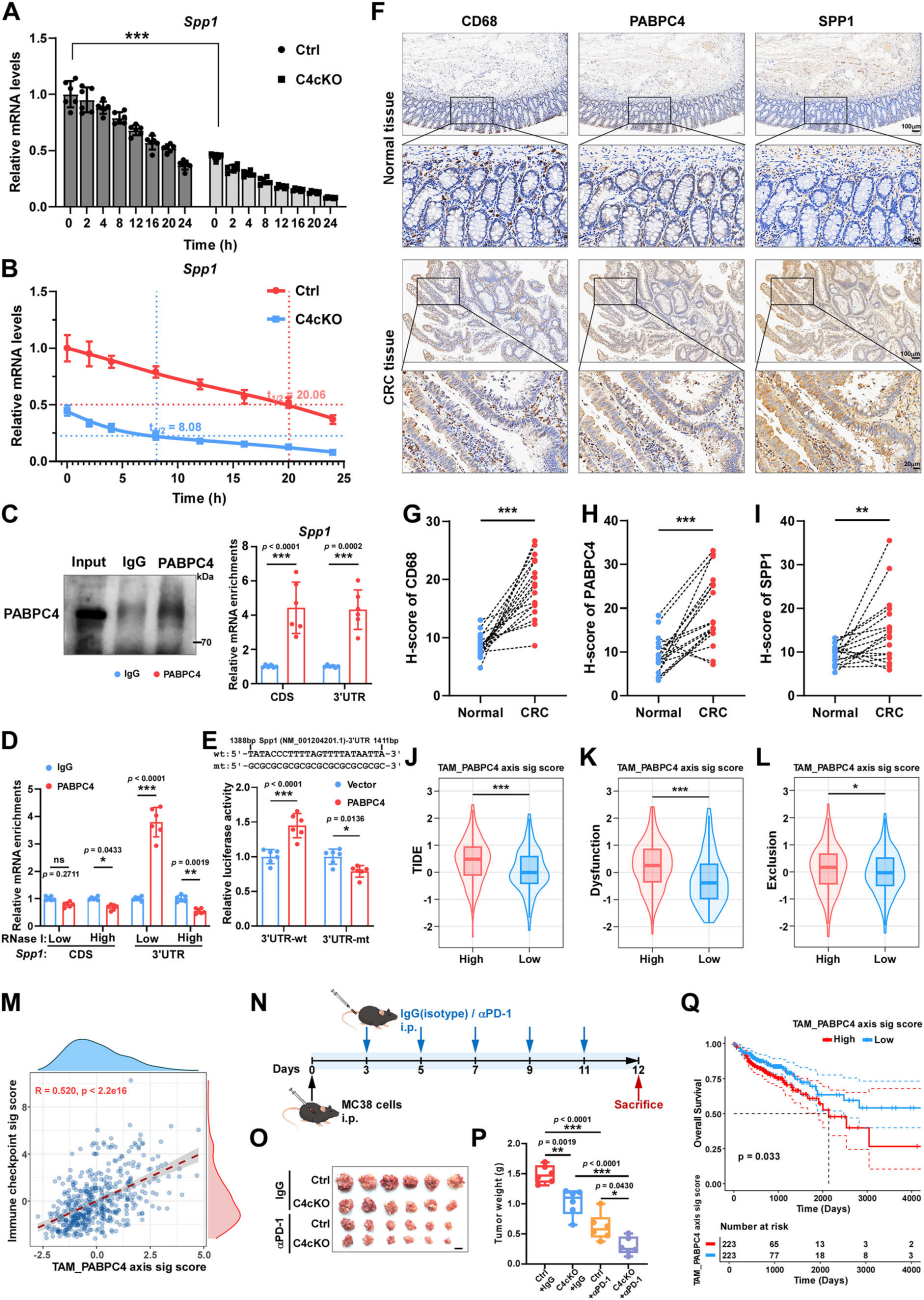
Macrophage PABPC4 stabilizes *SPP1* mRNA and correlates with immunotherapy resistance and unfavorable prognosis in CRC. (A) qPCR analysis of *Spp1* mRNA levels in PMs isolated from control (*Pabpc4* ^fl/fl^, Ctrl) and *Pabpc4* conditional knockout (*Pabpc4* ^fl/fl^; *Lyz2*‐Cre, C4cKO) mice (*n* = 6 per group). Cells were exposed to ActD (10 µg/mL), with cell lysates harvested at predefined time points (0 h, 2 h, 4 h, 8 h, 12 h, 16 h, 20 h, and 24 h). (B) mRNA decay curves and calculated half‑life of *Spp1* transcripts in Ctrl and C4cKO PMs derived from (A). Half‑life was defined as the time required for *Spp1* mRNA levels to decline to 50% of the respective 0 h control. (C) RIP assay. Left: western blotting confirming immunoprecipitation of PABPC4 protein from macrophage lysates. Right: qPCR analysis of *Spp1* mRNA enrichment in the CDS and 3′UTR regions of anti‐PABPC4 immunoprecipitates relative to IgG control, expressed as fold enrichment over IgG (*n* = 6 per group). (D) CLIP‐qPCR analysis of *Spp1* mRNA enrichment in the CDS and 3′UTR regions of anti‐PABPC4 immunoprecipitates following fragmentation with low (1:500) or high (1:50) concentrations of RNase I (*n* = 6 per group). (E) Dual‐luciferase reporter assay of wild‐type (wt) and mutant (mt) *Spp1* 3′UTR reporter plasmids upon co‐transfection with a *Pabpc4* overexpression plasmid (PABPC4) versus an empty vector control (Vector) (*n* = 6 per group). (F–I) Representative IHC staining (F) and corresponding H‐score quantification of CD68 (G), PABPC4 (H), and SPP1 (I) in human CRC tissues and matched adjacent normal tissues (*n* = 17 per group). Scale bars: 100 µm and 20 µm. (J–L) Tumor Immune Dysfunction and Exclusion (TIDE) algorithm analysis. Comparison of TIDE score (J), T cell dysfunction (K), and T cell exclusion (L) between CRC patient groups stratified by high versus low TAM_PABPC4 axis signature score. (M) Correlation analysis between the TAM_PABPC4 axis signature score (based on *CD68*, *LYVE1*, *PABPC4*, and *SPP1*) and a composite immune checkpoint gene signature score (including *CTLA4*, *CD274*, *PDCD1*, *PDCD1LG2*, *LAG3*, and *TIGIT*) in the TCGA‐COAD cohort. Pearson correlation coefficient is shown. (N) Schematic illustration of the in vivo anti‐PD‐1 treatment strategy. Ctrl and C4cKO mice bearing intraperitoneal MC38 tumors were injected intraperitoneally with IgG isotype control (IgG) or anti‐PD‐1 neutralizing antibody (αPD‐1) at a dose of 200 µg/mouse/injection. (O, P) Representative images (O) and quantified tumor weights (P) of peritoneal MC38 tumors harvested from Ctrl and C4cKO mice upon anti‐PD‐1 or IgG control treatment (*n* = 6 per group). Scale bar, 1 cm. (Q) Kaplan‐Meier overall survival curves of CRC patients stratified by high versus low median TAM_PABPC4 axis signature score in TCGA‐COAD cohort (*n* = 223 per group). Data are shown as mean ± SD. Statistical analyses were performed with unpaired two‐tailed Student's *t*‐test (A, C), two‑way ANOVA (D, E), paired two‐tailed Student's *t*‐test (G‐I), Wilcoxon rank‐sum test (J‐L), one‑way ANOVA (P), or log‐rank test (Q). ns stands for not significant, ^*^ *p* < 0.05, ^**^ *p* < 0.01, ^***^ *p* < 0.001.

We next assessed the clinical relevance of the macrophage PABPC4‐SPP1 axis in human CRC. Immunohistochemical analysis of sequential tissue sections confirmed coordinated upregulation of the macrophage marker CD68, along with PABPC4 and SPP1 in tumors versus matched normal counterparts (Figure 7F–I; Figure S10A). Consistently, macrophage abundance was significantly increased in COAD tumors with higher expression of the macrophage PABPC4‐SPP1 axis (Figure S10B). To quantitatively evaluate pathway activity, we derived a TAM_PABPC4‐SPP1 axis score based on the weighted expression of *CD68*, *LYVE1*, *PABPC4*, and *SPP1*. Subsequent analysis using the TIDE algorithm showed that high TAM_PABPC4‐SPP1 axis activity was associated with T cell dysfunction and an immunosuppressive microenvironment (Figure 7J–L; Figure S10C). This score also strongly correlated with the immune checkpoint signature score (Figure 7M). Analysis based on The Cancer Immunome Atlas (TCIA)‐derived immunophenoscore (IPS) indicated that elevated activity of the TAM_PABPC4‐SPP1 axis correlated with poor response to immune checkpoint blockade (Figure S10D). Moreover, in vivo experiments demonstrated that C4cKO potentiated anti‐PD‐1 efficacy (Figure 7N–P; Figure S10E–G). Critically, high activity of the macrophage PABPC4‐SPP1 axis emerged as a significant predictor of unfavorable overall survival in CRC patients (Figure 7Q). Collectively, these results suggest that the macrophage PABPC4‐SPP1 axis is clinically associated with potential immunotherapy resistance and poor prognosis in CRC.

## 4 Discussion

Although RBPs are recognized as critical contributors to cancer hallmarks, serving as potential prognostic indicators and novel therapeutic targets [7, 8, 9], their precise functions and mechanisms in regulating the TME are still largely elusive. This study identifies a previously unrecognized post‐transcriptional pathway that orchestrates immunosuppression in CRC. We demonstrate that the RNA‐binding protein PABPC4 is aberrantly upregulated in macrophages within the CRC TME, where it directly associates with the *SPP1* 3′UTR to stabilize its transcript, thereby reinforcing an immunosuppressive M2‐like activation of tumor‐associated macrophages. The macrophage PABPC4‐SPP1 axis notably impairs CD8^+^ T cell effector activity, accelerating tumor progression (Figure 8).

**FIGURE 8:**
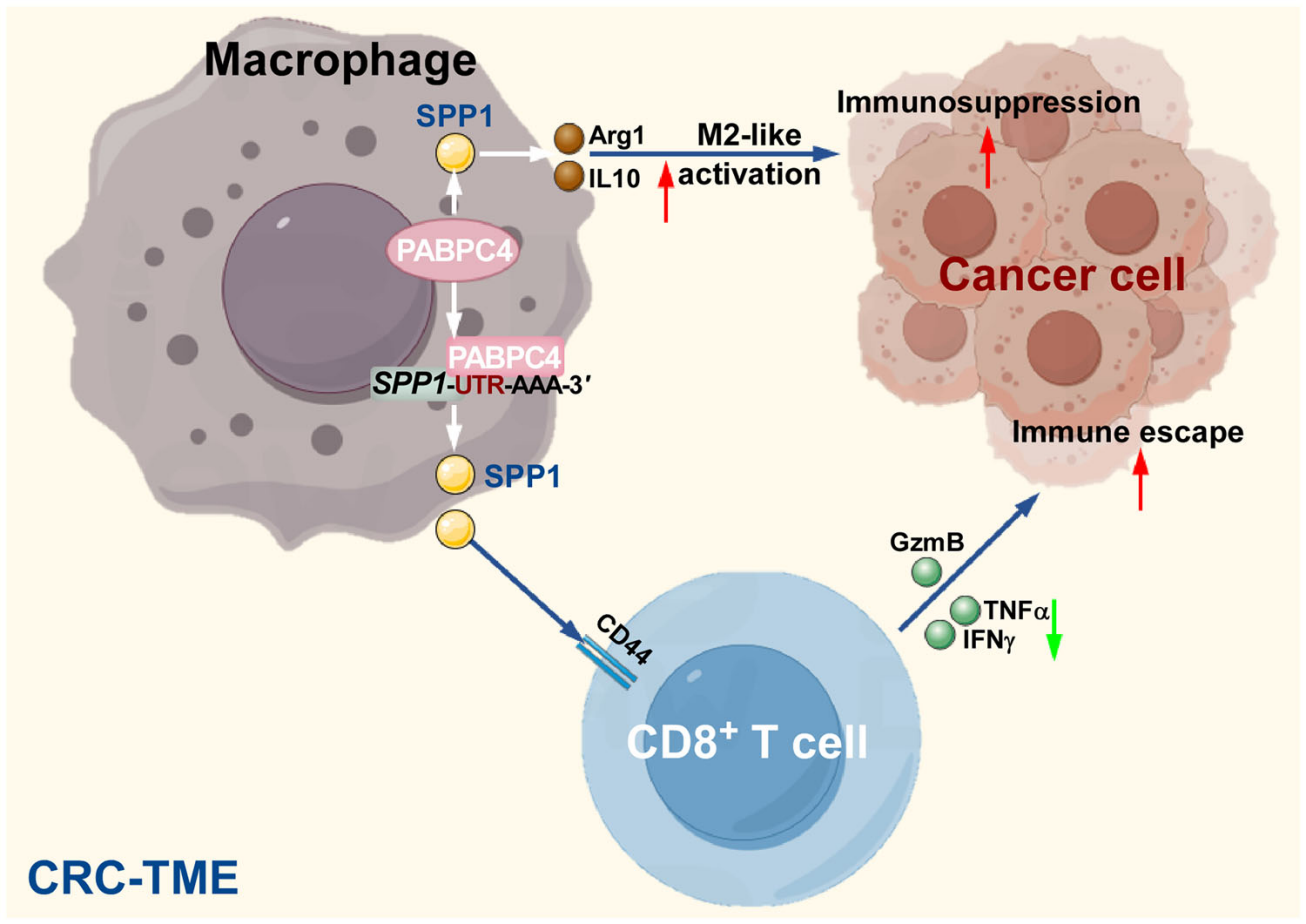
Proposed mechanism whereby macrophage *PABPC4* deficiency alleviates immunosuppression and suppresses CRC progression. In the colorectal cancer tumor microenvironment, macrophage PABPC4 stabilizes *SPP1* mRNA via 3′UTR binding, thereby sustaining M2‑like immunosuppressive macrophage polarization and, through the CD44 receptor, suppressing CD8^+^ T cell effector molecule expression. This coordinated process establishes an immunosuppressive niche and drives tumor progression.

Previous evidence indicates that the abnormal expression and dysfunction of PABPs, including PABPC1 [29, 30, 31], PABPC3 [14, 32], PABPC4 [33, 34], and PABPN1 [16, 35], can contribute to various oncogenic processes; however, their functions within other cellular components of the TME are largely unexplored. Here, we identify PABPC4 as the PABP family member most strongly associated with poor prognosis in CRC and demonstrate that its elevated expression within the TME originates primarily from macrophages. By employing myeloid‑specific conditional knockout mice in combination with macrophage depletion, CD8^+^ T cell blockade, and adoptive transfer of manipulated macrophages, we reveal that macrophage PABPC4 sustains TAM‐CD8^+^ T cell communication, fostering an immunosuppressive microenvironment that drives CRC progression. Macrophage PABPC4 ablation enhances antitumor immunity and restrains tumor progression in multiple preclinical tumor models. Notably, the consistent protumor phenotype observed in both melanoma and ovarian cancer settings suggests that this function of macrophage PABPC4 may not be strictly limited to colorectal cancer. We also acknowledge two key limitations of the current study. First, thioglycollate‐elicited PMs and in vitro differentiated BMDMs do not fully recapitulate the ontogeny and functional states of bona fide tumor‐associated macrophages in vivo. Second, the lack of efficacy of PABPC4 deficiency against CRC liver metastasis can be most plausibly attributed to the unique biological features of the hepatic microenvironment. The blunted response in the liver may stem from incomplete Cre‐mediated recombination in Kupffer cells, a technical constraint that aligns with previously reported lower *Lyz2* (*Lysm*) expression in this specific myeloid population [36]. This would leave the PABPC4‐SPP1 axis functionally unperturbed and capable of supporting metastatic progression. Moreover, the intrinsically immunosuppressive and tolerogenic nature of the liver microenvironment may create a “threshold effect”, whereby the modest anti‐tumor immunity unleashed by C4cKO alone is insufficient to overcome the pre‐existing, highly robust immunosuppressive network.

To elucidate the mechanism by which macrophage PABPC4 orchestrates immunosuppression, we integrated omics analyses with experimental validation and identified SPP1 as a pivotal downstream effector. Previous studies have established that macrophage‐derived SPP1 reinforces its immunosuppressive phenotype by upregulating anti‐inflammatory cytokines such as IL10 and TGFβ, promoting M2 polarization, and inhibiting macrophage apoptosis [25, 37, 38]. In contrast, we demonstrated that ablation of PABPC4, the SPP1 inducer we identified, did potentiate macrophage necroptosis and suppress M2‐like activation, whereas reconstitution of SPP1 expression effectively reverses these processes.

Moreover, SPP1 suppresses CD8^+^ T cell infiltration and effector function through multiple mechanisms, including engagement of CD44 or A2AR receptors on T cells, as well as suppression of cGAS‐STING and STAT1 signaling within macrophages [26, 38, 39, 40]. In our study, AAV‐mediated SPP1 restoration in vivo effectively reverses the macrophage polarization, CD8^+^ T cell activity, and tumor progression regulated by *Pabpc4* knockout. Furthermore, ligand‐receptor contribution analysis identified CD44 as the receptor contributing most significantly to SPP1 signaling, and blockade of CD44 signaling significantly attenuated the activation of CD8^+^ T cells induced by downregulation of the macrophage PABPC4‐SPP1 axis. Specifically, through combined RNA immunoprecipitation, mRNA decay assays, CLIP‐qPCR, and luciferase reporter assays, we discovered that PABPC4 enforces SPP1 expression by directly binding to its 3′UTR to stabilize its transcript, revealing a post‑transcriptional regulatory mechanism previously unrecognized in TAM‑mediated immunosuppression. Importantly, elevated activity of the macrophage PABPC4‑SPP1 axis correlates with immunosuppressive phenotypes, potential immunotherapy resistance, and poor clinical outcomes in CRC patients, nominating it as a compelling therapeutic target.

## 5 Conclusion

This work uncovers a critical link between post‑transcriptional gene regulation and cancer immunity, mediated by the macrophage PABPC4‐SPP1 axis. This pathway not only provides a mechanistic foundation for TAM‑driven CD8^+^ T cell dysfunction but also represents a promising target for enhancing anti‑tumor immunity in colorectal cancer and potentially other malignancies.

## Author Contributions

H.M. and S.H. designed and supervised this study. M.W., F.Y., Y.G. and L.L. executed the majority of experiments and analyses. Y.H., H.Y. and T.Z. contributed to the breeding and genotyping of gene‐edited mice. S.Z., H.H. and J.X. assisted in animal experiments. K.Z. and Y.X. supported data acquisition and interpretation. W.X. collected clinical samples. The manuscript was drafted by M.W., S.H. and H.M., and finalized with input from all authors.

## Conflicts of Interest

The authors declare no conflicts of interest.

## Supporting information




**Supporting File 1**: advs77361‐sup‐0001‐SuppMat.docx.

**Supporting File 2**: advs77361‐sup‐0002‐TablesS1‐S2.docx.

**Supporting File 3**: advs77361‐sup‐0003‐SupFig.docx.

## Data Availability

All data supporting the findings of this study are included within the main manuscript and its publicly accessible supplementary materials. Raw RNA sequencing data have been deposited in Genome Sequence Archive with accession CRA039011. Any other relevant data can be obtained from the corresponding author upon request.
