# Supplementary material for: Macrophage PABPC4‐SPP1 Axis Orchestrates Immunosuppression in Colorectal Cancer
Source: Adv Sci (Weinh). 2026 Aug 21;13(63):e77361. doi: 10.1002/advs.77361 (PMC13496150; doi:10.1002/advs.77361)
Supplement: Supplementary file 1 [file ADVS-13-e77361-s001.docx]

**Supplementary Tables**

**Table S1. Primer sequences used for qPCR.**

| **Genes** | **Forward（5′-3′）** | **Reversed（5′-3′）** |
| --- | --- | --- |
| *Pabpc4* | CTAAGGGCTATGCTTTTGTTCACT | ATCTACCCACGAACACTTTACGG |
| *Sirpa* | CGATCACCCGAAACCATACCG | CCACACCGATGAAGACATTCCAG |
| *Siglec-10* | CTCTAGAATGAGCACACAAAGGAC | TGGCCTTGCTGAACTTCCAGA |
| *Pd1* | TGGCTTCTAGAGGTCCCCAA | TTCAGCATAAGATCCTCCGACCA |
| *Pdl1* | GATCATCCCAGAACTGCCTGCAA | TCTTCAACGCCACATTTCTCCAC |
| *Lrp-1* | CTTCACGGTGCCTGATATCGAC | ATAAATGCCCTTTTGATGGCTTG |
| *Slamf7* | ATGACACAATCCCTTACACGGAA | GAGCTGGGACTCTTTACCACT |
| *Mac-1* | ATTCAGCCCCAAGAAAGTAGCAA | CTGGATATCTCCTTCGCGCAGA |
| *IL1β* | ACTCATTGTGGCTGTGGAGA | TTGTTCATCTCGGAGCCTGT |
| *IL6* | TCGTGGAAATGAGAAAAGAGTTG | AGTGCATCATCGTTGTTCATACA |
| *TNFa* | CTGAGGTCAATCTGCCCAAGTAC | CTTCACAGAGCAATGACTCCAAAG |
| *Arg1* | AGCCAAAGTCCTTAGAGATTATCGG | CATCAACAAAGGCCAGGTCCC |
| *IL10* | GAGAAGCATGGCCCAGAAATC | CGCATCCTGAGGGTCTTCA |
| *TGFβ* | GACCCTGCCCCTATATTTGGA | CCGGGTTGTGTTGGTTGTAGA |
| *Spp1-CDS* | ATTCCAATGAAAGCCATGACCAC | TCGTCAGATTCATCCGAGTCCAC |
| *Spp1-3’UTR* | AGAAGAGGCAAAAACACAGTTCCT | CCTGCTTAACCCTCACTAACACTT |
| *β-actin* | ACATCCGTAAAGACCTCTATGCC | ACCGATCCACACAGAGTACTTGC |

**Table S2. The antibodies used in flow cytometry in this study.**

| **Antibodies** | **Source** | **Identifier** |
| --- | --- | --- |
| Zombie Green™ Fixable Viability Kit | Biolgend | Cat# 423111 |
| Zombie Red™ Fixable Viability Kit | Biolgend | Cat# 423109 |
| Zombie UV™ Fixable Viability Kit | Biolgend | Cat# 423107 |
| Alexa Fluor® 700 anti-mouse CD45 | Biolgend | Cat# 103128; RRID: AB_493715 |
| PerCP/Cyanine5.5 anti-mouse CD45 | Biolgend | Cat# 103132; RRID: AB_893340 |
| APC/Cyanine7 anti-mouse F4/80 | Biolgend | Cat# 123118; RRID: AB_893477 |
| FlTC anti-mouse F4/80 | Biolgend | Cat# 123108; RRID: AB_893502 |
| Alexa Fluor® 700 anti-mouse CD90.2 (Thy1.2) | Biolgend | Cat# 105320; RRID: AB_493725 |
| FITC anti-mouse CD90.2 (Thy1.2) | Biolgend | Cat# 105306; RRID: AB_313177 |
| PE anti-mouse CD11c | Biolgend | Cat# 117308; RRID: AB_313777 |
| APC anti-mouse CD206 (MMR) | Biolgend | Cat# 141708; RRID: AB_10900231 |
| FlTC anti-mouse/human CD11b | Biolgend | Cat# 101206; RRID: AB_312789 |
| Pacific Blue™ anti-mouse Ly-6G/Ly-6C (Gr-1) | Biolgend | Cat# 108430; RRID: AB_893556 |
| PE anti-mouse Ly-6C | Biolgend | Cat# 128008; RRID: AB_1186132 |
| APC anti-mouse Ly-6G | Biolgend | Cat# 127614; RRID: AB_2227348 |
| PE anti-mouse CD3 | Biolgend | Cat# 100206; RRID: AB_312663 |
| ***Continued*** |  |  |
| **Antibodies** | **Source** | **Identifier** |
| APC/Cyanine7 anti-mouse CD4 | Biolgend | Cat# 100414; RRID: AB_312699 |
| PerCP/Cyanine5.5 anti-mouse CD8a | Biolgend | Cat# 100734; RRID: AB_2075238 |
| APC anti-mouse IFN-γ | Biolgend | Cat# 505810; RRID: AB_315404 |
| Brilliant Violet 510™ anti-mouse TNF-α | Biolgend | Cat# 506339; RRID: AB_2563127 |
| Brilliant Violet 421™ anti-human/mouse Granzyme B Recombinant | Biolgend | Cat# 396414; RRID: AB_2810603 |
| Brilliant Violet 605™ anti-mouse CD279 (PD-1) | Biolgend | Cat# 135219; RRID: AB_ 11125371 |
