# Supplementary material for: Macrophage PABPC4‐SPP1 Axis Orchestrates Immunosuppression in Colorectal Cancer
Source: Adv Sci (Weinh). 2026 Aug 21;13(63):e77361. doi: 10.1002/advs.77361 (PMC13496150; doi:10.1002/advs.77361)
Supplement: Supplementary file 3 [file ADVS-13-e77361-s003.docx]

**Supplementary figures**

**Figure S1. Expression landscape and prognostic significance of PABP family members in human cancers**

**(A)** mRNA expression levels of *PABPC4* across pan-cancer datasets from TCGA cohorts, analyzed using the TIMER online database.

**(B)** Protein expression levels of PABPC4 across pan-cancer datasets from the CPTAC cohort, analyzed using the UALCAN online database.

**(C-F)** Differential mRNA expression of *PABPC1* (C), *PABPC3* (D), *PABPC5* (E) and *PABPN1* (F) in CRC and matched normal tissues from TCGA-COAD and TCGA-READ cohorts, analyzed using the GEPIA2 online database.

**(G-J)** Kaplan-Meier survival curves of COAD and READ patients stratified by high versus low quartile expression of *PABPC1* (G), *PABPC3* (H), *PABPC5* (I) and *PABPN1* (J) in the TCGA cohort.

Statistical analyses were performed with Wilcoxon rank-sum test (A), one-way ANOVA (C-F) or log-rank test (G-J). **p* < 0.05, ***p* < 0.01, ****p* < 0.001.

**Figure S2. PABPC4 is aberrantly upregulated in macrophages and promotes tumor progression**

**(A)** Summary of the 20 clinical CRC specimens at different stages from the GSE178341 dataset used for single‑cell RNA‑seq analysis in this study.

**(B)** t‑SNE visualization of major cell clusters across different CRC stages.

**(C)** Heatmap of canonical markers for each identified clusters.

**(D)** Feature plot depicting *PABPC4* expression across major cell clusters at different CRC stages.

**(E)** Stacked bar chart of cell cluster abundance among CRC stages.

**(F)** t‑SNE visualization of macrophage subclusters.

**(G)** Stacked bar chart of macrophage subcluster abundance among CRC stages.

**(H)** t‑SNE visualization of macrophage subclusters at different CRC stages.

**(I)** Dot plot of markers for macrophage subclusters.

**(J)** Feature plot showing *PABPC4* expression across macrophage subclusters at different CRC stages.

**(K)** Violin plot showing normalized *PABPC4* expression levels in macrophages across different CRC stages.

**(L, M)** Representative images (L) and quantified tumor weights (M) of subcutaneous MC38 tumors harvested from control (*Pabpc4*^fl/fl^, Ctrl) and *Pabpc4* conditional knockout (*Pabpc4*^fl/fl^; *Lyz2*-Cre, C4cKO) mice (n = 5 per group). Scale bar, 1 cm.

**(N, O)** Gross images (N) and H&E staining (O) of liver metastasis from MC38 tumors in Ctrl and C4cKO mice (n = 4 per group). Scale bars, 1 cm (N), 100 μm (O).

**(P, Q)** Representative images (P) and quantified tumor weights (Q) of subcutaneous B16-F10 melanoma tumors harvested from Ctrl and C4cKO mice (n = 5 per group). Scale bar, 1 cm.

**(R)** Gross images (left) and bioluminescence imaging (right) of peritoneal ID8-LUC ovarian cancer metastases in Ctrl and C4cKO mice (n = 4 per group).

**(S)** Kaplan-Meier survival curves of ID8-LUC-bearing Ctrl and C4cKO mice (n = 12 per group).

**(T)** PCA plot of RNA‑seq datasets generated from Ctrl and C4cKO PMs subjected to 24 h of MC38-CM or vehicle control treatment (n = 3 per group).

Data are shown as mean ± SEM. Statistical analyses were performed with unpaired two‑sided Wilcoxon test (K), unpaired two‑tailed Student's t‑test (M, Q), or Gehan-Breslow-Wilcoxon test (S). **p* < 0.05, ***p* < 0.01.

**Figure S3:** **Flow cytometry gating strategy.**

**(A)** Gating strategy for identifying macrophage populations in tumor, adipose or spleen tissues. Macrophages were defined as live, single cells positive for CD45 and F4/80, and negative for CD90.2. Polarization states were defined as M1‑like (CD45^+^F4/80^+^CD11c^+^CD206^-^) and M2‑like (CD45^+^F4/80^+^CD11c^-^CD206^+^).

**(B)** Gating strategy for identifying myeloid‑derived suppressor cell (MDSC) populations. MDSCs were defined as live, single cells positive for CD11b and Gr1. Subsets were classified as polymorphonuclear MDSCs (PMN-MDSCs: CD11b^+^Ly6G^+^Ly6C^low^) and monocytic MDSCs (M‑MDSCs: CD11b^+^Ly6G^-^Ly6C^high^).

**(C)** Gating strategy for identifying T cell populations. T cells were defined as live, single cells positive for CD45 and CD3. Subsets were defined as CD4^+^ (CD45^+^CD3^+^CD4^+^CD8^-^) and CD8^+^ (CD45^+^CD3^+^CD4^-^CD8^+^) T cells. The frequencies of IFNγ^+^, TNFα^+^, and GzmB^+^ cells were quantified within the respective T cell subsets.

**Figure S4. Macrophage PABPC4 knockout remodels the immunosuppressive niche in adipose tissue of mice with peritoneal carcinomatosis**

**(A-D)** Flow cytometry plots (A) and quantitative analysis of total macrophages (B), M1‑like (C), M2‑like (D) subsets in visceral adipose tissue of MC38‑bearing control (*Pabpc4*^fl/fl^, Ctrl) and *Pabpc4* conditional knockout (*Pabpc4*^fl/fl^; *Lyz2*-Cre, C4cKO) mice (n = 5 per group).

**(E-H)** Representative plots (E) and quantitative analysis of total MDSCs (F), M-MDSCs (G), and PMN-MDSCs (H) in the same adipose tissue samples (n = 5 per group).

**(I-L)** Flow cytometry plots (I) and quantification of total T cells (J), CD4^+^ (K), and CD8^+^ (L) subsets in visceral fat tissue from Ctrl and C4cKO mice (n = 5 per group).

**(M-R)** Frequencies of IFNγ^+^ (M, N), TNFα^+^ (O, P), and GzmB^+^ (Q, R) cells within total, CD4^+^, and CD8^+^ T cells (n = 5 per group).

Data are mean ± SD. Statistical analyses were performed with unpaired two‑tailed Student's t‑test (B-D, F-H, J-L, N, P, R). **p* < 0.05, ****p* < 0.001.

**Figure S5. Impact of macrophage PABPC4 knockout on immune cell infiltration in the spleen of mice with peritoneal CRC**

**(A-D)** Flow cytometry plots (A) and quantitative analysis of total macrophages (B), M1‑like (C), and M2‑like (D) subsets in spleen of MC38‑bearing control (*Pabpc4*^fl/fl^, Ctrl) and *Pabpc4* conditional knockout (*Pabpc4*^fl/fl^; *Lyz2*-Cre, C4cKO) mice (n = 5 per group).

**(E-H)** Representative plots (E) and quantitative analysis of total MDSCs (F), M-MDSCs (G), and PMN-MDSCs (H) in the same spleen samples (n = 5 per group).

**(I-L)** Flow cytometry plots (I) and quantification of total T cells (J), CD4^+^ (K), and CD8^+^ (L) subsets in spleen from Ctrl and C4cKO mice (n = 5 per group).

**(M-R)** Frequencies of IFNγ^+^ (M, N), TNFα^+^ (O, P), and GzmB^+^ (Q, R) cells within total, CD4^+^, and CD8^+^ T cells (n = 5 per group).

Data are mean ± SD. Statistical analyses were performed with unpaired two‑tailed Student's t‑test (B-D, F-H, J-L, N, P, R). **p* < 0.05, ***p* < 0.01, ****p* < 0.001.

**Figure S6. Macrophage PABPC4 ablation remodels the immunosuppressive microenvironment in subcutaneous CRC tumors**

**(A-D)** Flow cytometry plots (A) and quantitative analysis of total macrophages (B), M1‑like (C), and M2‑like (D) subsets in subcutaneous tumors from MC38‑bearing control (*Pabpc4*^fl/fl^, Ctrl) and *Pabpc4* conditional knockout (*Pabpc4*^fl/fl^; *Lyz2*-Cre, C4cKO) mice (n = 5 per group).

**(E-H)** Representative plots (E) and quantitative analysis of total MDSCs (F), M-MDSCs (G), and PMN-MDSCs (H) in the same tumor tissues (n = 5 per group).

**(I-L)** Flow cytometry plots (I) and quantification of total T cells (J), CD4^+^ (K), and CD8^+^ (L) subsets in the same tumor tissues (n = 5 per group).

**(M-P)** IFNγ^+^ frequencies within total (N), CD4^+^ (O), and CD8^+^ (P) T cells (n = 5 per group).

Data are mean ± SD. Statistical analyses were performed with unpaired two‑tailed Student's t‑test (B-D, F-H, J-L, N-P). **p* < 0.05, ***p* < 0.01, ****p* < 0.001.

**Figure S7. Macrophage PABPC4 regulates T cell activation, intercellular communication and promotes tumor progression through SPP1-dependent mechanisms**

**(A, B)** GSEA plots showing variation trend of the GOBP_T_CELL_ACTIVATION (A) and GOBP_T_CELL_ACTIVATION_INVOLVED_IN_IMMUNE_RESPONSE (B) signatures in C4cKO PMs relative to controls.

**(C)** Bubble plot depicting significantly upregulated ligand-receptor pairs involved in macrophage-NK and CD8^+^ T cell communication in stage IV CRC compared to normal tissues.

**(D)** Heatmap visualization of the cellular interaction roles within the SPP1 signaling pathway‑mediated cell‑cell communication network in stage IV CRC.

**(E, F)** Representative plots (E) and quantitative analysis of total macrophages (F) in peritoneal MC38 tumors harvested from control (*Pabpc4*^fl/fl^, Ctrl) and *Pabpc4* conditional knockout (*Pabpc4*^fl/fl^; *Lyz2*-Cre, C4cKO) mice with or without AAV‑mediated Spp1 restoration (n = 5 per group).

**(G, H)** Representative plots (G) and quantitative analysis of CD8^+^ T cells (H) from peritoneal tumors (n = 5 per group).

**(I, J)** Adoptive transfer model of peritoneal MC38 tumors. Representative images (I) and tumor weights (J) of mice co‑injected with MC38 cells and PMs derived from Ctrl or C4cKO mice that were transduced with lentivirus overexpressing Spp1 (OE) or negative control vector (NC) (n = 6 per group). Scale bar, 1 cm.

**(K, L)** Adoptive transfer model of subcutaneous MC38 tumors. Representative images (K) and tumor weights (L) of mice co‑injected with MC38 cells and PMs derived from Ctrl or C4cKO mice transduced with Spp1‑OE or NC lentivirus (n = 5 per group). Scale bar, 1 cm.

**(M, N)** Adoptive transfer model of subcutaneous B16‑F10 tumors. Representative images (M) and tumor weights (N) of mice co‑injected with B16‑F10 cells and PMs derived from Ctrl or C4cKO mice transduced with Spp1‑OE or NC lentivirus (n = 5 per group). Scale bar, 1 cm.

**(O)** Adoptive transfer model of peritoneal ID8 tumors. Representative gross images of mice co‑injected with ID8‑LUC cells and PMs derived from Ctrl or C4cKO mice transduced with Spp1‑OE or NC lentivirus (n = 4 per group). Scale bar, 1 cm.

Data are mean ± SD. Statistical analyses were performed with two-way ANOVA (F, H, J, L, N). **p* < 0.05, ***p* < 0.01, ****p* < 0.001.

**Figure S8. Macrophage PABPC4-SPP1 axis suppresses CD8^+^ T cell immune activity via CD44 receptor signaling**

**(A)** Bar plot showing the relative contribution of ligand‑receptor pairs driving enhanced communication strength of the SPP1 signaling pathway in stage IV CRC.

**(B-I)** In vitro co‑culture assay. Peritoneal macrophages (PMs) isolated from control (*Pabpc4*^fl/fl^, Ctrl) or *Pabpc4* conditional knockout (*Pabpc4*^fl/fl^; *Lyz2*-Cre, C4cKO) mice were co‑cultured with activated CD8^+^ T cells (1:5, 72 h) upon IgG isotype control or anti-CD44 neutralizing antibody (40 ng/mL) treatment. Frequencies of IFNγ^+^ (B, C), TNFα^+^ (D, E), GzmB^+^ (F, G) and PD-1^+^ (H, I) cells in CD8^+^ T cells (n = 5 per group).

Data are mean ± SD. Statistical significance was assessed via two-way ANOVA (C, E, G, I). **p* < 0.05, ****p* < 0.001.

**Figure S9.** **PABPC4 enhances macrophage susceptibility to necroptosis via SPP1 signaling**

**(A)** GSEA plots showing alterations in glycolysis, OXPHOS, protein processing in endoplasmic reticulum, apoptosis, and necroptosis pathways in peritoneal macrophages (PMs) following *Pabpc4* knockout (upper panel), and changes in the same pathways following *Pabpc4* knockout under MC38‑conditioned medium treatment for 24 h (lower panel).

**(B)** Glucose uptake levels in PMs derived from Ctrl (*Pabpc4*^fl/fl^) and C4cKO (*Pabpc4*^fl/fl^; *Lyz2*-Cre) mice (n = 6 per group).

**(C)** Lactate production levels in Ctrl and C4cKO PMs (n = 6 per group).

**(D)** ATP levels in Ctrl and C4cKO PMs (n = 6 per group).

**(E)** Reactive oxygen species (ROS) levels in Ctrl and C4cKO PMs (n = 3 per group).

**(F)** Heatmap of significantly differentially expressed genes involved in necroptosis pathway in PMs following *Pabpc4* knockout.

**(G)** Western blot analysis of necroptosis-related proteins in Ctrl and C4cKO PMs transduced with Spp1‑OE or NC lentivirus, followed by 24 h of MC38‑CM treatment.

**(H, I)** Dead cell percentages in Ctrl and C4cKO PMs after Spp1‑OE or NC lentiviral transduction and 24 h MC38‑CM treatment, shown as representative cytometric plots (H) and quantitative analysis (I) (n = 6 per group).

Data are mean ± SD. Statistical analyses were performed with unpaired two-tailed Student's t-test (B-E) or two-way ANOVA (I). **p* < 0.05, ****p* < 0.001.

**Figure S10. Clinical relevance of the macrophage PABPC4-SPP1 axis**

**(A)** IHC analysis of CD68, PABPC4, and SPP1 in human CRC versus matched normal tissues. Scale bar, 100 μm.

**(B)** Immune infiltration levels in the TCGA-COAD cohort stratified by high versus low TAM_PABPC4 axis signature score.

**(C)** Tumor Immune Dysfunction and Exclusion (TIDE) algorithm analysis. Comparison of *CD274* score between CRC patient groups stratified by high versus low TAM_PABPC4 axis signature score.

**(D)** Immunophenoscore (IPS) analysis predicting anti‑PD‑1/CTLA‑4 response in CRC patients stratified by TAM_PABPC4 axis signature score.

**(E)** Schematic illustration of the in vivo anti-PD-1 treatment strategy. Control (*Pabpc4*^fl/fl^, Ctrl) and *Pabpc4* conditional knockout (*Pabpc4*^fl/fl^; *Lyz2*-Cre, C4cKO) mice bearing subcutaneous MC38 tumors were intraperitoneally administered either IgG isotype control (IgG) or anti-PD-1 neutralizing antibody (αPD-1) at a dose of 200 μg/mouse/injection.

**(F, G)** Representative images (F) and quantified tumor weights (G) of subcutaneous MC38 tumors harvested from Ctrl and C4cKO mice upon anti-PD-1 or IgG control treatment (n = 5 per group). Scale bar, 1 cm.

Data are mean ± SD. Statistical analyses were performed with Wilcoxon rank-sum test (B, C), two‑way ANOVA (D) or one‑way ANOVA (G). **p* < 0.05, ***p* < 0.01, ****p* < 0.001.
