# Supplementary material for: Macrophage PABPC4‐SPP1 Axis Orchestrates Immunosuppression in Colorectal Cancer
Source: Adv Sci (Weinh). 2026 Aug 21:e77361. Online ahead of print. doi: 10.1002/advs.77361 (PMC13496150; doi:10.1002/advs.77361)

Uncropped western blots with the indicated areas of selection

Figure 1H - PABPC4

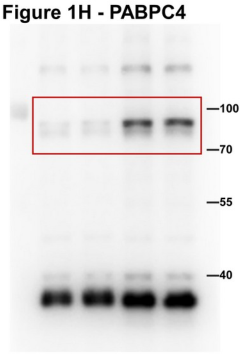

Figure 1H - β-actin

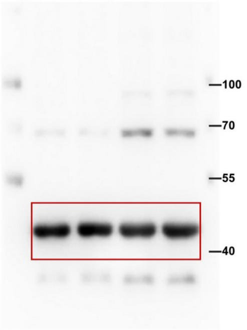

Figure 1K - PABPC4

Figure 1K - β-actin

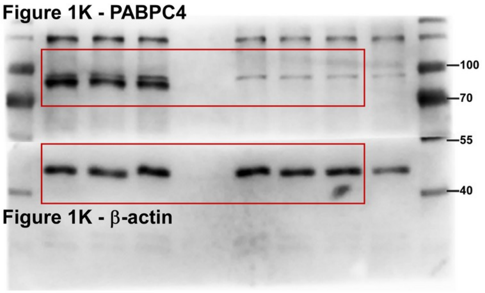

Figure 6D - PABPC4

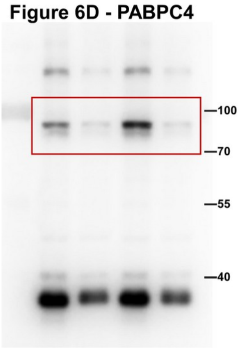

Figure 6D - SPP1

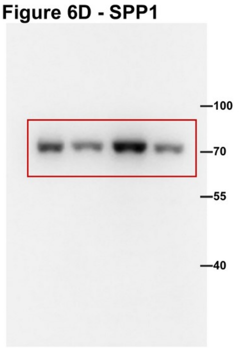

Figure 6D - β-actin

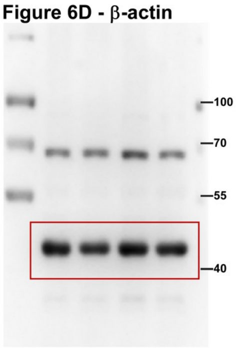

Figure 7C - PABPC4

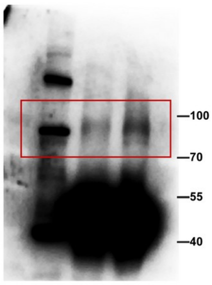

Figure S9 - SPP1

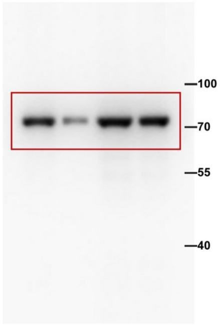

Figure S9 - STAT1

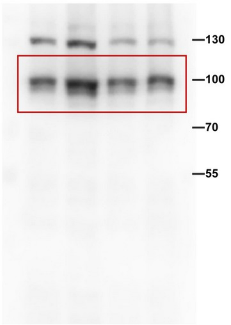

Figure S9 - p-STAT1 (Y701)

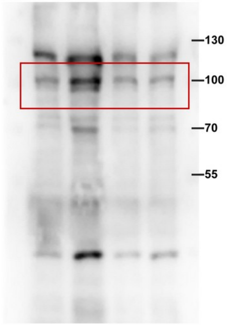

Figure S9 - p-Stat1 (S727)

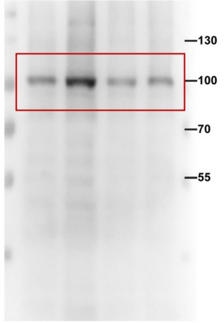

Figure S9 - p-MLKL (S345)

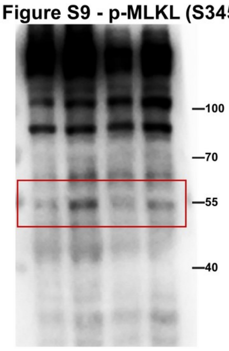

Figure S9 - p-RIPK1 (S166)

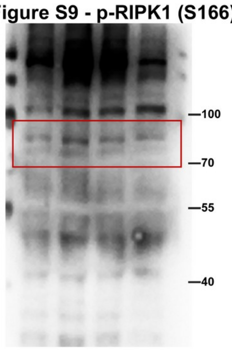

Figure S9 - p-RIPK3 (S232)

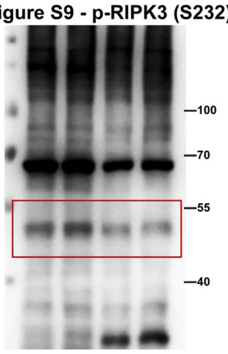

Figure S9 - β-actin

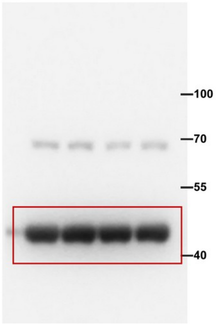

Supplement: Supplementary file 1 [file ADVS-9999-e77361-s001.docx]
